# AI Literacy and Training Needs Among French Midwifery Students: National Cross-Sectional Online Survey and Exploratory Qualitative Follow-Up

**DOI:** 10.2196/94310

**Published:** 2026-10-26

**Authors:** Sylvain Kotzki, Sonia Knepfler, Lionel Di Marco, Nicolas Vuillerme

**Affiliations:** 1 Centre de Recherche en Santé Intégrée, Université Grenoble Alpes, CNRS, Grenoble INP, LIG Sangria, 6 Chemin Saint-Ferjus, La Tronche, Grenoble, 38700, France, 33 476637119; 2 Department of Midwifery, Université de Strasbourg, Strasbourg, France; 3 Department of Midwifery, Centre Hospitalier Universitaire Grenoble Alpes, Grenoble, France; 4 Institut Universitaire de France, Paris, France

**Keywords:** midwifery education, AI, AI literacy, digital health, health professions education, cross-sectional survey, curriculum

## Abstract

**Background:**

AI is increasingly relevant to health care and health professional education, but AI-related knowledge, perceptions, and training needs among midwifery students remain underdocumented. Evidence is needed to inform midwifery curricula and to clarify how students understand and operationalize AI in training and placements.

**Objective:**

This cross-sectional online study aimed to assess AI-related knowledge, perceptions, perceived risks, and training needs among French midwifery students.

**Methods:**

We conducted a national cross-sectional online survey of students enrolled in years 2 to 5 of the French midwifery curriculum between January and May 2025, complemented by exploratory qualitative follow-up interviews. Of 34 midwifery schools and universities contacted, 30 confirmed dissemination of the survey link through institutional and student-association channels. The voluntary, anonymous survey combined 5 previously published instruments or questionnaires administered in linguistically validated French versions. Likert-type items were analyzed descriptively on a 1 to 5 scale at the item level and through post hoc descriptive thematic summaries across 5 domains and 23 themes. Analyses were restricted to fully completed survey entries, with no imputation, inferential testing, or subgroup comparisons. Eight volunteer students from 1 school in eastern France participated in semistructured interviews, which were analyzed thematically to provide exploratory contextual observations.

**Results:**

Of 414 initiated survey entries, 190 (45.9%) were fully completed and analyzed. Self-reported AI knowledge and familiarity were below the scale midpoint, with the lowest score observed for familiarity with advanced AI techniques (1.20, 95% CI 1.13‐1.27). General AI familiarity was higher (n=91, 47.9% agreement) than familiarity with specific concepts such as machine learning (n=20, 10.5%) or deep learning (n=13, 6.8%). Perceived ability to use AI for clinical purposes was limited (2.05, 95% CI 1.93‐2.16). In contrast, students strongly endorsed AI education, particularly the belief that students and professionals should be trained in AI (4.11, 95% CI 4.02‐4.20). Prior exposure to AI (1.36, 95% CI 1.27‐1.45) and perceived institutional and social support (1.43, 95% CI 1.34‐1.52) were low. Ethics- and safety-related concerns were salient, with the highest score for evidence and safety requirements (4.04, 95% CI 3.96‐4.12). Exploratory interviews suggested that students often referred to AI through everyday generative tools, reported little supervised exposure to AI in clinical placements, and emphasized safety, privacy, accountability, and preservation of human judgment.

**Conclusions:**

Interpreted as a time-bound baseline, these findings suggest an AI readiness gap among French midwifery students, in which low perceived knowledge and limited clinical readiness coexist with strong demand for training and high salience of safety and governance. They support the progressive and proportionate introduction of AI literacy in midwifery education, explicitly distinguishing general AI, generative AI tools, and regulated clinical AI systems. Future work should develop and validate midwifery-specific AI literacy measures and evaluate curriculum interventions.

## Introduction

AI is increasingly relevant to health care, from diagnostic support to monitoring, prediction, documentation, and decision support [1,2]. In some narrow tasks, such as image-based classification, AI systems can reach levels of performance comparable to those of clinicians, although their broader use in care continues to raise questions about safety, transparency, accountability, and clinical validation [2,3]. For future clinicians, AI therefore represents not only a technological development but also an educational challenge: students need to understand what these systems do, when their outputs may or may not be trusted, and what professional responsibilities remain when AI tools are used in care [4]. Current evidence suggests that health professions students’ knowledge, perceptions, and training needs regarding AI are heterogeneous. Students often express interest in AI and recognize its potential relevance for health care, while also reporting limited familiarity with core concepts, limited formal training, and concerns about reliability, professional responsibility, and the preservation of human judgment [5,6]. At present, however, AI-related competencies in health curricula remain heterogeneous, and structured training is still uneven despite emerging competency and curriculum frameworks [7,8].

In France, a mandatory digital health core curriculum was introduced for health students in 2022, and the 2024 reform of the midwifery curriculum incorporated digital health competencies; however, AI-specific learning outcomes remain limited or insufficiently explicit [9,10]. A further difficulty is that the term “AI” covers different realities that students may not clearly distinguish. General AI familiarity refers to broad awareness of AI as a field and its associated vocabulary. Generative AI tools, such as general-purpose conversational assistants, are widely available consumer applications that students may already use for study, writing, or clinical-placement tasks [11]. Regulated or clinically deployed AI systems, by contrast, are tools intended for use in care and are subject to clinical validation, professional governance, and regulatory oversight. These categories are easily conflated: familiarity with consumer generative AI tools may be mistaken for an understanding of clinically deployed AI systems. This ambiguity is important when interpreting students’ self-reported AI knowledge, readiness, and training needs.

These questions are particularly relevant to midwifery education. AI applications are being explored in women’s and perinatal health, including prenatal diagnostic support, monitoring, and risk prediction, although their current role in routine midwifery practice remains limited [12-14]. Midwifery is also distinctive because it is grounded in continuity of care, clinical judgment, and direct relationships with women and families. These features may shape how midwifery students perceive technologies that could affect communication, trust, and shared decision-making. Qualitative work with practicing midwives has reported both interest in the potential of AI and concerns about its effects on empathy, trust, and ethical decision-making in childbirth [15]. Authors have therefore argued that AI education should be integrated into nursing and midwifery training so that these professionals can contribute to safe, critical, and patient-centered use of AI [16-18]. However, midwives and midwifery students remain underrepresented in existing AI perception and readiness studies, which have focused mainly on medical students and physicians in countries such as Canada, the United States, and Turkey [19-21]. In addition, available instruments were mostly developed for medical students or broader health professional populations, and few data are available on how these domains apply to midwifery students. To our knowledge, no national study has examined AI-related knowledge, perceptions, risks, and training needs among midwifery students in France.

This study aimed to assess AI-related knowledge, perceptions, perceived risks, and training needs among French midwifery students through a national cross-sectional online survey, complemented by exploratory qualitative interviews to contextualize selected findings. The survey drew on previously published instruments or questionnaires assessing AI-related perceptions, readiness, learning intentions, and perceived risks in health professions education.

## Methods

### Study Design

We conducted a national cross-sectional online survey of midwifery students in France, complemented by an exploratory qualitative follow-up strand based on semistructured individual interviews. The survey provided the primary quantitative data. The qualitative strand was used to contextualize and illustrate selected survey findings. Given its exploratory, single-site, small-sample nature, no claim of data saturation is made. The study was conducted over 5 months, from January to May 2025.

### Setting, Population, and Recruitment

The target population comprised all students enrolled in midwifery programs in France during the 2024 to 2025 academic year. In France, access to midwifery studies occurs after a first year of higher education through national health-studies entry pathways, either a health-specific access year (PASS [Specific Health Access Pathway]) or a health-access option within a university bachelor’s program (L.AS). The midwifery-specific curriculum begins only after admission to the midwifery school; eligibility was therefore restricted to students enrolled in years 2 to 5 of the midwifery curriculum.

Program directors and faculty leads from 34 midwifery schools were contacted. Thirty institutions confirmed dissemination of the survey link to their students through institutional mailing lists and learning management platforms (n=30, 88.2%). We used voluntary convenience sampling through institutional and student association channels. To support completion, the survey was disseminated in several waves during the data collection period, and up to 2 reminders were sent through the same institutional and student networks. Partial responses were automatically saved so that participants could resume the questionnaire later. Participation was voluntary and uncompensated. The distribution of respondents by institution is provided in Multimedia Appendix 1.

### Survey Instruments

The survey combined 5 previously published instruments or questionnaires with heterogeneous levels of prior psychometric evaluation, assessing health care students’ perceptions, readiness, learning intentions, and perceived risks regarding AI in medicine [21-25]. The Medical Artificial Intelligence Readiness Scale for Medical Students (MAIRS-MS) by Karaca et al [21] is a formally developed and psychometrically validated scale. The questionnaire by Li et al [25] is grounded in the Theory of Planned Behavior and was evaluated using confirmatory factor analysis and structural equation modeling. The questionnaire by Boillat et al [23] was systematically developed from a literature review and reported internal consistency and factor adequacy indices. By contrast, the questionnaires by Sit et al and Park et al [22,24] were previously published survey instruments with more limited psychometric evidence.

The full French versions were administered as previously translated, culturally adapted, and linguistically validated, without modification to item wording, response options, or item order [26]. This previous work established conceptual and linguistic equivalence in French; however, the psychometric properties of these French versions in French-speaking midwifery students specifically remain to be established.

In total, the survey comprised 112 items: 94 Likert-type items and 18 additional items, including 13 single-choice and 5 open-ended questions. Likert responses were harmonized onto a 1 to 5 scale, with higher scores indicating stronger agreement or endorsement. Additional items captured sociodemographic characteristics, including age group, gender, year of study, training institution, and region, as well as prior exposure to AI, generative AI tools, AI-related training, and digital health.

### Survey Administration

The survey was hosted on a secure online platform and was open between January and May 2025. It was organized into 5 pages per screen, beginning with sociodemographic items followed by AI-related sections. An introductory page presented the study aims, voluntary participation, data-protection measures, and the estimated completion time. No directly identifying information was collected.

The survey link was disseminated in several waves, with up to 2 reminders sent through institutional contacts and student associations. The link was not technically individualized. To preserve complete anonymity, no mechanism to prevent duplicate entries was implemented. In practice, access was restricted to midwifery students because the link was transmitted locally by school administrations to their own students. Participants could complete the survey on any internet-enabled device. Partial responses were saved automatically to allow later completion, but only fully completed questionnaires were retained for analysis. Of 414 questionnaires initiated, 190 (45.9%) were fully completed. We report this online survey in accordance with the CHERRIES guidelines (Checklist 1).

### Qualitative Follow-Up Strand

The exploratory qualitative follow-up strand consisted of semistructured individual interviews conducted with volunteer midwifery students from a school in eastern France. The site was selected pragmatically because of local access within the research network. This recruitment strategy is reported transparently and is addressed as a limitation. Students were invited during teaching sessions and by email through a local coordinator, with attention paid to variation in year of study, gender, and prior exposure to AI or digital health.

Interviews were conducted by a member of the research team who was a midwife. They took place either face-to-face in a quiet room at the school or via secure videoconference, lasted for a maximum of 30 minutes, and were audio-recorded with participants’ consent. Recordings were transcribed verbatim in French and anonymized by removing names, locations, and other potentially identifying information. An interview guide, informed by the literature, the 5 questionnaires, and preliminary survey findings, was used to support the interviews; it was not formally pilot-tested. The interview guide explored students’ self-reported knowledge of AI, prior AI-related training, personal and professional uses of AI, perceived future roles of AI in health care, barriers to AI use, digital skills, prior exposure to digital health, and perceptions of the future of health care in relation to digital technologies. When needed, a brief general definition of AI was provided to support a shared understanding during the interview. In total, 8 students were interviewed.

Qualitative data were analyzed using a pragmatic reflexive thematic approach informed by Braun and Clarke [27]. Analysis involved familiarization, initial coding, grouping of related codes, theme development and refinement, and review of the thematic structure against the study questions. Given the exploratory scope and the limited number of interviews, coding was managed manually in a structured spreadsheet rather than dedicated qualitative-analysis software. Descriptive codes were assigned by S Knepfler, and S Kotzki independently reviewed the coding framework and thematic structure. Discrepancies were discussed until consensus, and the codebook was refined iteratively. Analytic memos and reflexive notes were maintained. To support trustworthiness, we used investigator triangulation during coding, maintained an audit trail of coding decisions, and compared themes across participants and training years. Member checking was not conducted.

For descriptive characterization of the interview participants, an additional post hoc participant-level categorization of self-reported AI knowledge was performed after completion of the interview series. This categorization was distinct from the reflexive thematic analysis described above. The 6 cognitive levels of the original Bloom taxonomy (knowledge, comprehension, application, analysis, synthesis, and evaluation) were used as an interpretive framework [28]. The interview guide did not present these categories to participants, and participants did not self-select a Bloom level. S Knepfler made the initial assignment for each participant based on the interview material relating to their reported AI knowledge and understanding; S Kotzki independently reviewed the assignments, and discrepancies were resolved through discussion until consensus. A separate category, “No self-reported AI knowledge,” which is not part of Bloom taxonomy, was added for participants who reported no AI knowledge. No formal scoring instrument was administered. The resulting categories were descriptive, researcher-assigned interpretations of self-reported material and should not be interpreted as an objective assessment of AI knowledge.

### Data Management and Statistical Analysis

Survey responses from the 5 instruments were merged into a single analytic dataset and first examined at the item level within each original questionnaire. For each Likert-type item, we computed response frequencies, percentages, and weighted means on the 1 to 5 scale, SD, SE, and 95% CI, calculated as mean ± 1.96 × SE. Analyses were descriptive only. No inferential statistics, hypothesis tests, *P* values, significance thresholds, or normality testing were performed. Analyses were restricted to fully completed questionnaires (complete-case analysis, n=190), with no imputation of missing data. Quantitative analyses were conducted in Python 3.11 using pandas and NumPy for data management and pingouin for Cronbach α and descriptive statistics. Subgroup comparisons were not prespecified, and no inferential subgroup analyses were performed. Although variables such as year of study, prior AI exposure, and previous AI-related training were available, the study was neither designed nor powered for between-group comparisons. In particular, the subgroups reporting prior AI-related training were small: 26 of 190 (13.7%) respondents reported having previously taken AI courses or training, and 17 of 190 (8.9%) reported having received AI teaching or training. Subgroup analyses were therefore considered but not conducted; this is addressed as a limitation.

### Item Aggregation and Thematic Domain Mapping

To support an integrated reading across the 5 instruments, we derived post hoc aggregated thematic scores. This aggregation was not based on a published conceptual framework and was not intended to create new validated scales. Instead, it was used as a descriptive, integrative analytic lens across questionnaires.

Two investigators (S Kotzki and S Knepfler) independently assigned each Likert-type item to 1 of 5 overarching domains: (1) current AI skills and knowledge, (2) practical application and clinical integration, (3) attitudes and perceptions, (4) training, learning, and commitment, and (5) ethical, regulatory, and risk-related aspects. Within these domains, items were grouped into 23 specific themes, plus a theme for sociodemographic characteristics. Discrepancies were resolved by discussion until consensus and documented in an item-to-theme matrix. No item wording, response option, item order, or original questionnaire was changed (Multimedia Appendix 1).

For each theme, a descriptive theme score was computed as the mean of its constituent Likert items on a 1 to 5 scale. To ensure that the associated 95% CI reflects participant-level sampling variability rather than dispersion across the theme’s constituent items, an individual theme score was first computed for each participant as the mean of that participant’s responses to the theme’s items; the theme-level SD, SE, and 95% CI (1.96 × SE) were then derived from these 190 participant-level scores. For the attitudes-and-perceptions composite (Cronbach *α*=0.48), the theme score is reported descriptively as mean and SD without a CI, given its conceptual heterogeneity (Multimedia Appendix 1). Cronbach α was used as an indicator of internal coherence for descriptive interpretation, not as evidence of formal scale validation. Themes with α values below conventional thresholds were interpreted cautiously, with item-total correlations and content coherence examined to assess whether the aggregation remained meaningful. The attitudes-and-perceptions domain showed low internal consistency and was therefore treated as a heterogeneous descriptive composite. A supplementary item-to-instrument-to-domain mapping table is provided to make the post hoc aggregation transparent (Multimedia Appendix 1).

### Ethical Considerations

This study was approved by the Ethics Committee for Integrity and Research in Health Professions Education of the International Francophone Society for Medical Education (notice 1210‐2023, approval date November 26, 2023). Participation was voluntary and uncompensated. At the beginning of the survey and before each interview, participants received information on the study aims, confidentiality safeguards, data protection, and their right to withdraw without consequences. Electronic consent was obtained before survey completion, and verbal consent was recorded at the start of each interview.

Survey data were collected anonymously and stored on secure institutional servers, with access restricted to the research team. Interview recordings and transcripts were pseudonymized, with identifying information removed or altered. Only deidentified data were analyzed. Deidentified datasets and analysis scripts may be made available from the corresponding author upon reasonable request, subject to applicable regulations and data-protection requirements.

We followed the STROBE (Strengthening the Reporting of Observational Studies in Epidemiology; quantitative survey), CHERRIES (Checklist for Reporting Results of Internet E-Surveys; web-based survey), and COREQ (Consolidated Criteria for Reporting Qualitative Research; qualitative strand) reporting guidelines. Selected survey patterns and exploratory interview observations are summarized in a descriptive contextualization matrix (Multimedia Appendix 1). Completed checklists are provided in Checklist 1.

## Results

### Participation and Respondent Characteristics

Of the 34 institutions contacted, 30 (88.2%) confirmed dissemination of the survey link to their students. In total, 414 questionnaires were initiated, and 190 (45.9%) were fully completed and retained for analysis. The 224 (54.1%) noncompleted entries included 41 (9.9%) with no response beyond the landing page. Among the 183 partially completed entries, attrition occurred mainly at the beginning of the questionnaire: most stopped on the first of the survey’s 5 pages (n=122, 66.7%), and only 6 (3.3%) reached the first AI-related item, indicating that most noncompleters did not progress beyond the sociodemographic section. Respondent characteristics are summarized in Table 1; respondents were predominantly women and distributed across years 2 to 5, with the institutional distribution detailed in Multimedia Appendix 1.

**Table 1.** Characteristics of participants in the quantitative and qualitative strands.

Characteristics	Quantitative strand (N=190), n (%)	Qualitative strand (N=8), n (%)
Gender		
Women	183 (96.3)	8 (100)
Men	4 (2.1)	0 (0)
Prefer not to answer	3 (1.6)	^a^—
Age (y)		
<20	26 (13.7)	0 (0)
20‐24	137 (72.1)	5 (62.5)
25‐29	15 (7.9)	0 (0)
30‐34	6 (3.2)	1 (12.5)
>34	6 (3.2)	1 (12.5)
Not reported	0 (0)	1 (12.5)
Year of study		
2nd	47 (24.7)	0 (0)
3rd	55 (29.0)	2 (25)
4th	60 (31.6)	4 (50)
5th	27 (14.2)	2 (25)
Not recorded	1 (0.5)	0 (0)
Post hoc researcher-assigned categorization of self-reported AI knowledge^b^		
No self-reported AI knowledge	Not assessed	2 (25)
Knowledge	Not assessed	4 (50)
Comprehension	Not assessed	2 (25)
Application	Not assessed	0 (0)
Analysis	Not assessed	0 (0)
Synthesis	Not assessed	0 (0)
Evaluation	Not assessed	0 (0)

a Not applicable.

b This categorization was used only for descriptive characterization of the qualitative participants. Categories were assigned post hoc from interview material after completion of the interview series, using the 6 cognitive levels of the original Bloom taxonomy as an interpretive framework [28]; participants were not asked to select a Bloom level. “No self-reported AI knowledge” is an additional category outside Bloom taxonomy. The categorization was researcher assigned and was not an objective assessment of AI knowledge. This variable was not assessed in the quantitative survey strand.

The exploratory qualitative follow-up strand comprised 8 volunteer students from a single midwifery school (Table 1). They were in the third to fifth years of training and varied in age and prior exposure to digital tools; no second-year students and no men students participated. These interviews are reported below as single-site, exploratory contextual observations and are not intended to be representative of the survey population.

To summarize patterns across the 5 questionnaires, item-level results were also grouped into post hoc descriptive thematic summaries (5 domains and 23 themes), reported here as descriptive theme scores on the 1 to 5 scale rather than as validated scale scores (see *Methods* section and Multimedia Appendix 1).

### AI-Related Knowledge and Familiarity

Self-reported AI skills and knowledge were below the scale midpoint of 3 across all related themes, ranging from 1.20 (95% CI 1.13‐1.27) for familiarity with advanced AI techniques to 2.89 (95% CI 2.78‐3.00) for analytical concepts in AI for health (Figure 1). Foundational items were uneven: agreement was higher for basics statistics (109/190, 57.4%) than for named AI methods. A notable item-level contrast was observed between general AI familiarity (91/190, 47.9% agreement) and familiarity with specific concepts such as machine learning (20/190, 10.5%) and deep learning (13/190, 6.8%; Figure 1; Multimedia Appendix 1).

**Figure 1.**
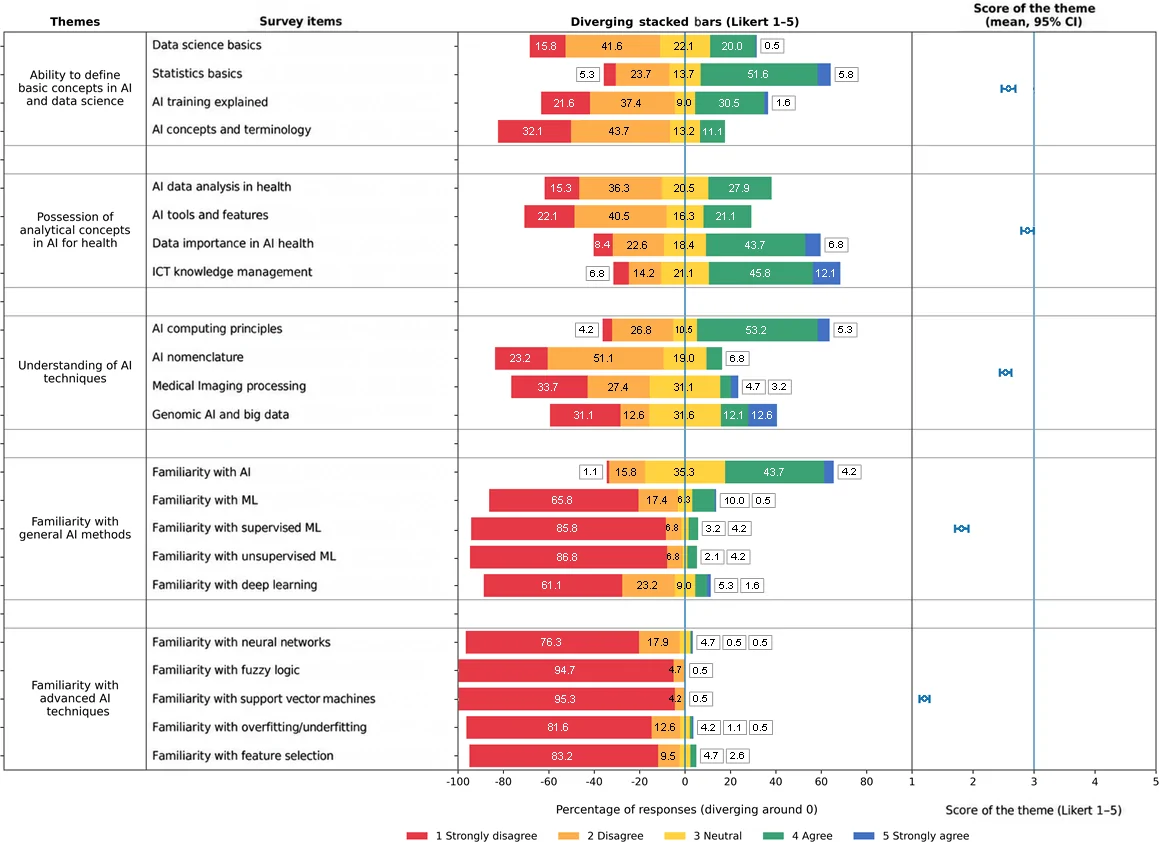
Current AI skills and knowledge (n=190). This figure shows the distribution of responses to survey items (Likert scale 1‐5) and the associated theme scores. The right-hand panel shows, for each theme, the theme score (mean) with its 95% CI as a dot plot; the vertical reference line indicates the neutral value (3). Labels under the survey items column have been translated and shortened for readability; the full original French wording of each item is available in Multimedia Appendix 1. ICT: information and communication technology; ML: machine learning.

In the interviews, students described their AI knowledge in broad terms and referred mainly to everyday examples or general-purpose generative tools; they rarely named or explained specific methods, and formal AI instruction was described as rare or fragmented. These accounts provided exploratory, illustrative context for the low self-reported familiarity seen in the survey.

### Perceived Ability to Use AI in Practice

Perceived ability to use AI in practice was generally limited (Figure 2). The theme score for professional use was closest to the midpoint (3.13, 95% CI 3.01‐3.25), whereas clinical use (2.05, 95% CI 1.93‐2.16) and perceived productivity gains (1.99, 95% CI 1.84‐2.14) were below it. At the item level, difficulty was frequently reported for selecting AI tools for clinical problems (121/190, 63.7%, disagreement) and for explaining AI tools to patients (126/190, 66.3%, disagreement; Figure 2).

**Figure 2.**
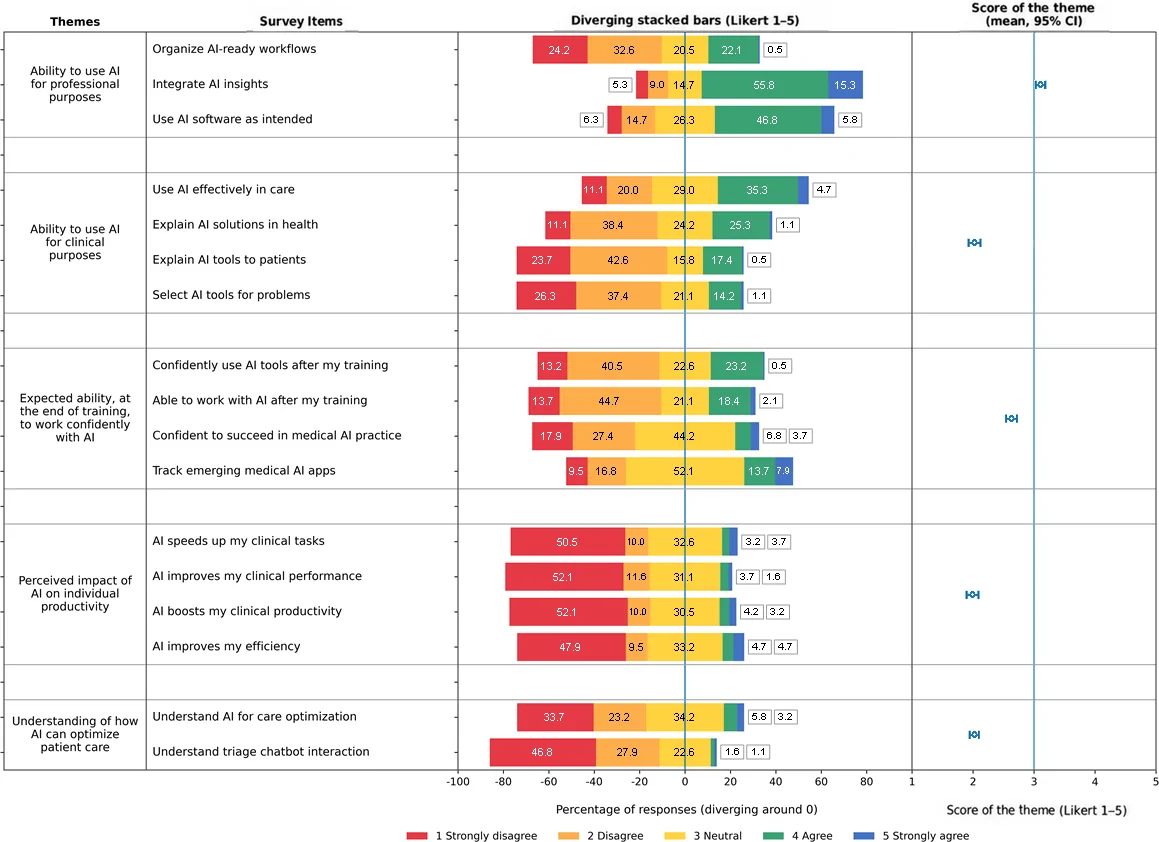
Perceived ability to use AI in practice (n=190). This figure shows the distribution of responses to survey items (Likert scale 1‐5) and the associated theme scores. The right-hand panel shows, for each theme, the theme score (mean) with its 95% CI as a dot plot; the vertical reference line indicates the neutral value (3). Labels under the survey items column have been translated and shortened for readability; the full original French wording of each item is available in Multimedia Appendix 1.

In the interviews, students described little exposure to AI during clinical placements; existing digital systems were generally not described as AI, and generative tools, when used, were mainly used outside patient care for academic purposes. These remarks are reported as exploratory contextual observations from the single-site strand.

### Perceived Impact of AI on Health Care and Professional Roles

Perceptions of AI’s broader impact were above the midpoint (descriptive composite theme mean score 3.50, SD 0.51; Figure 3). Agreement was high that AI would have a positive impact on medical practice during respondents’ careers (165/190, 86.8%) and that it would play an important role in health care (155/190, 81.6%). Views on more disruptive implications were more divided or neutral, including responses on reducing interest in medical imaging careers and on AI handling some specialties within respondents’ lifetimes (Figure 3).

**Figure 3.**
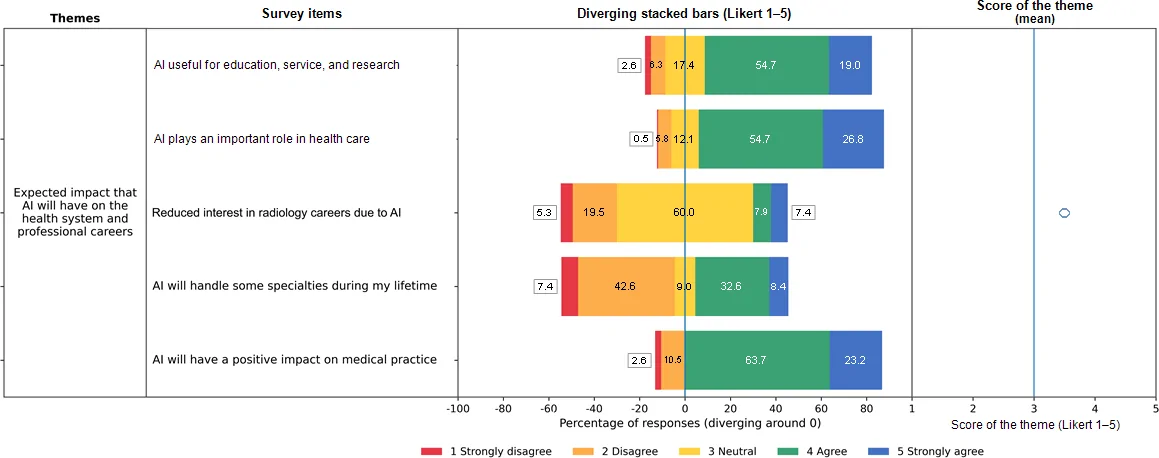
Perceptions of AI’s impact and professional projection (n=190). This figure shows the distribution of responses to survey items (Likert scale 1‐5) and the associated theme scores. The right-hand panel shows, for each theme, the theme score (mean) with its 95% CI as a dot plot, except for the attitudes-and-perceptions composite, which is reported descriptively without a 95% CI; the vertical reference line indicates the neutral value (3). Labels under the survey items column have been translated and shortened for readability; the full original French wording of each item is available in Multimedia Appendix 1.

In the interviews, students anticipated that AI would become part of their professional environment but found it difficult to specify how it would affect day-to-day midwifery practice. These accounts provided exploratory context for the survey responses.

### Training Needs, Prior Exposure, and Institutional Support

Endorsement of AI education was high (Figure 4). The highest descriptive theme score was observed for the belief that students and professionals should be trained in AI (4.11, 95% CI 4.02‐4.20), with high agreement that health students need AI education (169/190, 88.9%). Perceived need for further training was also high (3.69, 95% CI 3.59‐3.79). In contrast, perceived institutional and social support (1.43, 95% CI 1.34‐1.52) and prior exposure (1.36, 95% CI 1.27‐1.45) were among the lowest scores observed, and self-reported confidence (2.47, 95% CI 2.34‐2.59) and commitment (2.76, 95% CI 2.63-2.89) were modest (Figure 4).

**Figure 4.**
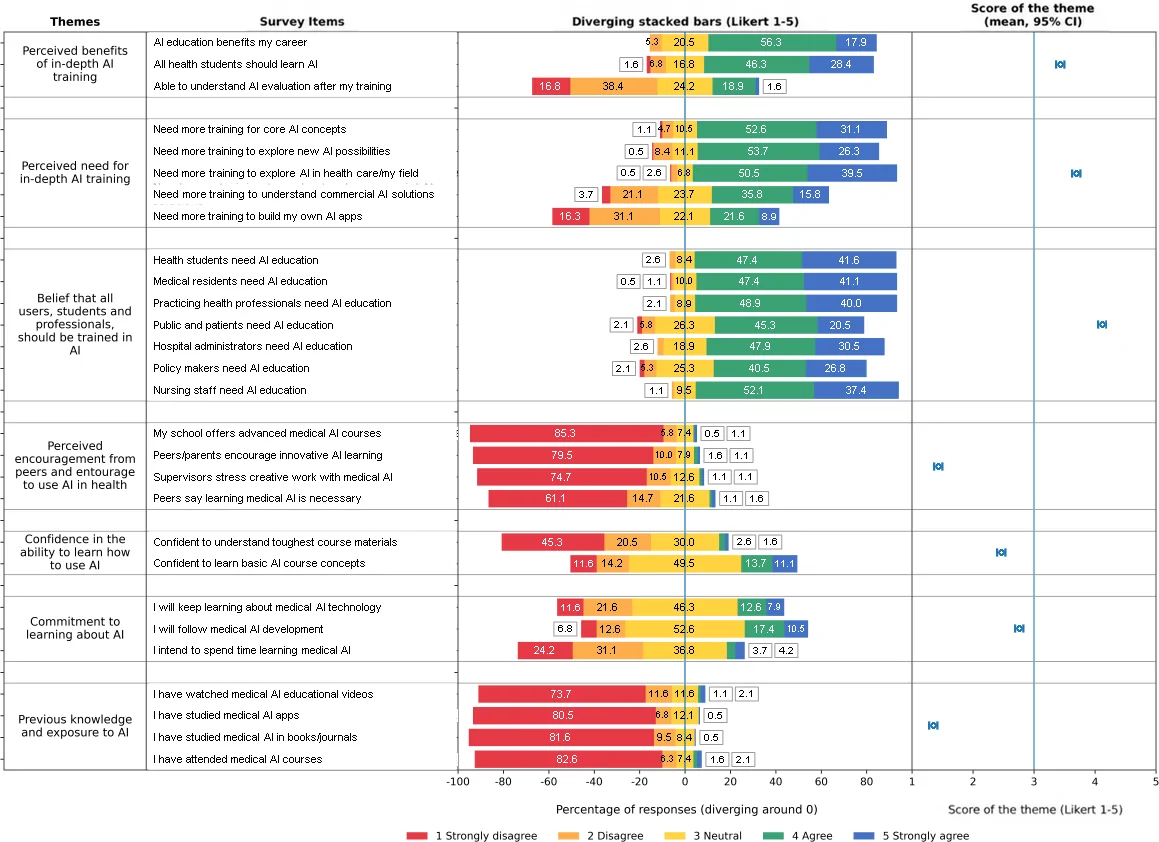
Training needs, prior exposure, and institutional support (n=190). This figure shows the distribution of responses to survey items (Likert scale 1‐5) and the associated theme scores. The right-hand panel shows, for each theme, the theme score (mean) with its 95% CI as a dot plot; the vertical reference line indicates the neutral value (3). In the interviews, students expressed interest in being better prepared for AI but described their learning as largely self-directed and reported limited access to structured teaching or supervised opportunities. These accounts illustrate the survey pattern of high reported demand alongside low reported support. Labels under the survey items column have been translated and shortened for readability; the full original French wording of each item is available in Multimedia Appendix 1.

### Ethical, Regulatory, and Risk-Related Perceptions

Ethical, regulatory, and risk-related themes were above the midpoint (Figure 5), with the highest descriptive score for evidence and safety requirements (4.04, 95% CI 3.96‐4.12) and a lower score for legal and ethical use of AI (3.25, 95% CI 3.15‐3.35). Data privacy was frequently identified as a key implementation issue (158/190, 83.2%). Perceptions of downstream risks varied: many anticipated dehumanization of care (154/190, 81.1%) and clinician skill erosion (144/190, 75.8%), whereas fewer agreed that AI would harm patients (53/190, 27.9%; Figure 5).

In the interviews, students raised data protection, accountability, and preservation of human judgment as priorities, while acknowledging limited knowledge of existing regulations. These exploratory remarks are reported for context only.

**Figure 5.**
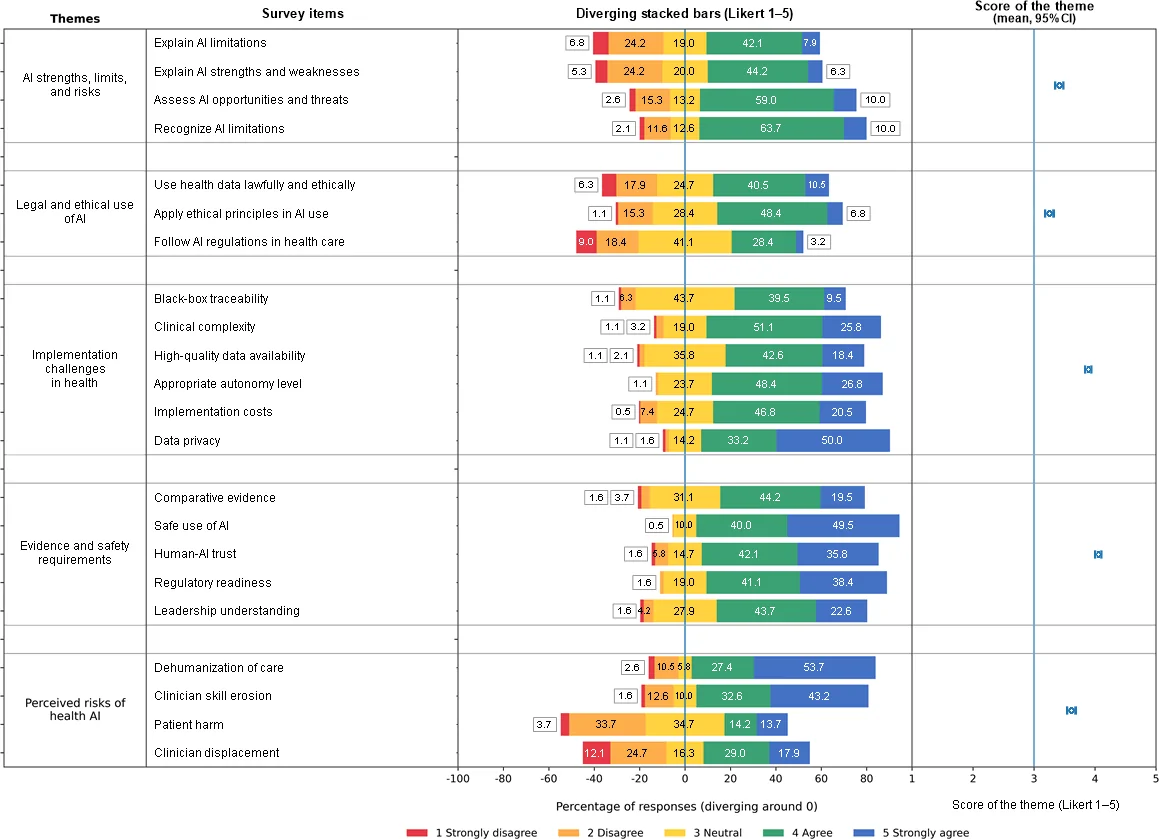
Ethical, regulatory, and risk-related perceptions (n=190). This figure shows the distribution of responses to survey items (Likert scale 1‐5) and the associated theme scores. The right-hand panel shows, for each theme, the theme score (mean) with its 95% CI as a dot plot; the vertical reference line indicates the neutral value (3). Labels under the survey items column have been translated and shortened for readability; the full original French wording of each item is available in Multimedia Appendix 1.

## Discussion

### Principal Findings

In this national cross-sectional online survey of French midwifery students, complemented by exploratory qualitative interviews, students reported low self-perceived AI knowledge and familiarity, particularly regarding specific AI concepts and methods, together with limited perceived ability to use AI in clinical practice. At the same time, they expressed broadly positive expectations about AI’s future role in health care while remaining uncertain about its concrete implications for midwifery practice. Demand for AI training was high, yet prior exposure and perceived institutional and social support were low. Students also placed strong emphasis on privacy, safety, accountability, and the preservation of human judgment, and they were concerned about the dehumanization of care and skill erosion. The exploratory interviews provided contextual illustrations of these patterns rather than independent confirmation of them.

### Interpretation and Comparison With Prior Work

The studies underpinning the 5 questionnaires used in this survey provide an initial point of comparison. Conducted in different national and educational contexts—including Turkey, the United Kingdom, the United States, China, and multiregional samples—and predominantly involving medical students, physicians, or broader health professions learners, these studies reported a recurrent pattern: favorable attitudes toward AI coexisting with limited familiarity with core concepts, limited formal training, perceived need for AI education, and ethical or professional concerns [21-25]. The same domains were prominent among French midwifery students, suggesting shared educational challenges across health professions while also highlighting the need for discipline-specific interpretation in midwifery education.

Recent evidence in health professions education points in the same direction. A systematic review of studies involving health students and academics across nursing, midwifery, medicine, dentistry, allied health, and psychology found that positive attitudes toward AI were commonly accompanied by limited AI literacy, limited formal training, and recurring ethical concerns; students also frequently identified universities as an appropriate setting for acquiring AI competencies [29]. Consistent with this, a Turkish survey of nursing and midwifery students found that most participants had not received AI education despite generally favorable attitudes toward AI [30]. Similarly, in Jordan, medical students and pathology trainees were often aware of AI, but formal exposure through medical schools remained limited, and respondents expressed a need for education on AI applications, risks, and ethical or legal implications [31].

For midwifery, these findings intersect with broader questions about how digital and AI-supported transformation affects maternity care. A qualitative synthesis of midwives’ experiences of digital health indicates that digital transformation influences professional roles, relational work, accountability, and digitally mediated communication [32]. Similar concerns appear in the health education literature, where midwifery students have emphasized that AI cannot substitute for empathy and relational care [29]. Evidence from nurse practitioner education also suggests that the educational value of clinical AI tools depends on adequate training and supervised exposure rather than on availability alone, with implications for diagnostic reasoning and confidence [33]. In this context, our findings indicate that French midwifery students may hold positive expectations about AI while reporting limited perceived readiness, limited supervised exposure, and ethical and professional concerns, including about safety, privacy, and the preservation of human judgment.

The contrast between self-reported familiarity with “AI” in general and lower familiarity with specific methods such as machine learning or deep learning also deserves attention. The interviews suggest that students may use the term “AI” to refer to different realities, including general AI, consumer generative AI tools, and regulated or clinically deployed AI systems. This distinction is increasingly relevant, given evidence that health professions students already use generative AI tools for study, writing, and clinical-placement tasks while receiving comparatively little formal instruction in them [11]. A survey of first-year Japanese medical students similarly found widespread generative AI use alongside less common formal learning and strong interest in structured generative AI literacy [34]. Familiarity with consumer generative tools may therefore inflate apparent AI familiarity without implying readiness to understand, evaluate, or use regulated clinical AI systems.

Because AI knowledge and familiarity were self-perceived, students may have overestimated or underestimated their actual understanding, and the ambiguity around “AI” may have affected responses in either direction. The findings should therefore be read as describing perceived, rather than demonstrated, knowledge and readiness.

### Implications for Midwifery Education and AI Literacy

These findings suggest that introducing AI literacy progressively within midwifery curricula could help align students’ interest in AI with their currently limited preparedness, although the present study does not itself validate any specific curriculum. For midwifery education, AI literacy should therefore not be limited to technical awareness. It should also help students consider how AI-supported information is communicated within the continuity of care, how professional responsibility is maintained in shared decision-making, and how human judgment and relational presence are preserved when digital outputs enter encounters with women and families. Teaching could explicitly distinguish general AI, generative AI tools, and regulated clinical AI systems and address data privacy, accountability, transparency, validation principles, human oversight, communication around AI-supported decisions, and safe-use boundaries for generative tools. This distinction is important because clinically deployed AI systems raise specific requirements related to safety assurance, procedural safeguards, transparency, human oversight, data governance, and professional accountability [35-37]. In Europe, these issues can also be linked pedagogically to the AI Act and to data-protection requirements under the General Data Protection Regulation (GDPR) [38,39]. Given students’ strong emphasis on safety and ethics, these elements may offer a useful entry point.

Because the instruments used here were generic rather than midwifery-specific and because the French versions underwent cross-cultural translation, adaptation, and linguistic validation but not psychometric validation among French-speaking midwifery students, a midwifery-specific AI literacy measure remains to be developed and validated [26]. Such work could combine qualitative exploration with educators, students, and clinicians, followed by consensus-building and psychometric validation.

### Strengths and Limitations

To the best of our knowledge, this is the first national study of AI-related knowledge, perceptions, perceived risks, and training needs among midwifery students in France. Conducted during the 2024 to 2025 academic year, it draws on multiple institutions and combines survey data with exploratory qualitative observations. It is intended to serve as a baseline for repeated assessments in future academic years, allowing changes in AI exposure, perceived readiness, and training needs to be monitored over time.

Several limitations should be considered. First, the survey was long, with 112 items across 5 pages. Of the 414 questionnaires initiated, 190 were completed, corresponding to a completion rate of 45.9% (190/414). Most attrition occurred early, before the AI-related sections. This pattern, together with voluntary participation, may introduce selection and nonresponse bias, and respondents may have differed from nonrespondents in their interest in or familiarity with AI. In addition, because no technical mechanism was used to prevent duplicate submissions in order to preserve complete anonymity, repeated participation by the same individual cannot be completely excluded.

Second, the data were collected between January and May 2025. Given the rapid evolution of AI and the uptake of generative AI in education and health care, the findings should be interpreted as a time-bound baseline rather than a stable estimate.

Third, no inferential subgroup analyses were prespecified or conducted, and the subgroups reporting prior AI courses or training were small (26/190 and 17/190). The data therefore do not support conclusions by year of study, prior exposure, or previous AI-related training. Fourth, the 5 domains and 23 themes were post hoc descriptive thematic summaries used as an integrative lens across questionnaires, not validated scales. Relatedly, the survey measures of AI knowledge and familiarity were self-reported rather than objectively assessed. In the qualitative strand, the participant-level categorization shown in Table 1 was a post hoc researcher interpretation of self-reported interview material rather than a participant self-rating or an objective assessment. Students may therefore have overestimated or underestimated their understanding, particularly because the term “AI” may have been interpreted differently among respondents.

Fifth, the qualitative component was an exploratory single-site follow-up strand with 8 volunteers. It included no men students and no second-year students; no claim of data saturation is made, and these observations contextualize but do not generalize beyond the participants. The absence of men participants in the qualitative sample should be interpreted in light of the gender composition of the profession and of our survey sample. In France, midwifery remains a predominantly female profession, with national DREES (Directorate for Research, Studies, Evaluation and Statistics) data indicating that 97% of practicing midwives were women in 2025 [40]. Men were also a small minority in our survey sample (4/190, 2.1%). The absence of men interviewees therefore remains a limitation of perspective, but should not be interpreted as evidence of gender-related differences.

### Conclusions

French midwifery students reported low perceived AI knowledge and limited perceived readiness to use AI in clinical practice, alongside strong demand for training and high salience of safety, privacy, accountability, and human judgment, in the context of low prior exposure and limited institutional support. Interpreted as a time-bound baseline, these findings suggest a readiness gap that is unlikely to be closed by informal, self-directed learning alone. They support the progressive and proportionate introduction of AI literacy in midwifery education in France, explicitly distinguishing general, generative, and clinical AI systems, and underline the need to develop and validate a midwifery-specific AI literacy measure to support future curriculum design and evaluation.

## Supplementary material

10.2196/94310 Multimedia Appendix 1 The distribution of respondents by midwifery school/university, the thematic grouping of survey items and internal consistency estimates, and the joint display integrating quantitative findings, illustrative quotes, and mixed methods inferences.

10.2196/94310 Checklist 1 STROBE (cross-sectional quantitative component), CHERRIES (web-based survey), and COREQ (exploratory qualitative follow-up strand).

## Abbreviations

CHERRIES: Checklist for Reporting Results of Internet E-Surveys
COREQ: Consolidated Criteria for Reporting Qualitative Research
DRESS: Directorate for Research, Studies, Evaluation and Statistics
GDPR: General Data Protection Regulation
MAIRS-MS: Medical Artificial Intelligence Readiness Scale for Medical Students
PASS: Specific Health Access Pathway
STROBE: Strengthening the Reporting of Observational Studies in Epidemiology
