# Supplementary material for: AI Literacy and Training Needs Among French Midwifery Students: National Cross-Sectional Online Survey and Exploratory Qualitative Follow-Up
Source: JMIR Med Educ. 2026 Oct 26;12:e94310. doi: 10.2196/94310 (PMC13706253; doi:10.2196/94310)
Supplement: Supplementary file 1 [file mededu-v12-e94310-s001.docx]

**Table S1. Responders distribution by French midwifery schools/universities**

| Midwifery Schools/Universities | Responders  n (%) |
| --- | --- |
| Strasbourg | 27 (14.2) |
| Grenoble | 18 (9.5) |
| Besançon | 14 (7.4) |
| Limoges | 11 (5.8) |
| Paris | 11 (5.8) |
| Montpellier | 11 (5.8) |
| Poitiers | 10 (5.3) |
| La Réunion | 8 (4.2) |
| Angers | 8 (4.2) |
| Brest | 8 (4.2) |
| Lille | 7 (3.7) |
| Nîmes | 7 (3.7) |
| Toulouse | 6 (3.2) |
| Nice | 5 (2.6) |
| Marseille | 5 (2.6) |
| Amiens | 4 (2.1) |
| Metz | 4 (2.1) |
| Lyon | 3 (1.6) |
| Sorbonne Université | 3 (1.6) |
| Dijon | 2 (1.1) |
| Versailles | 2 (1.1) |
| Rouen | 2 (1.1) |
| Puy-de-Dôme | 1 (0.5) |
| Caen | 1 (0.5) |
| Tahiti | 1 (0.5) |
| Colmar | 1 (0.5) |
| Rennes | 1 (0.5) |
| Martinique | 1 (0.5) |
| Tours | 1 (0.5) |
| Not reported | 7 (3.7) |

**Table S2. Item-to-instrument-to-domain mapping**

Only Likert-type items included in the post hoc descriptive thematic summaries are listed. Sociodemographic, categorical, and open-ended items were not included in theme-score calculations. Item short labels are abbreviated English paraphrases used for readability and traceability and do not replace the original item wording. When needed, item direction was harmonized for thematic scoring.

| Original Item | Original Instrument | French Administered Item | Item Short Label | Specific Theme | Aggregated Domain |
| --- | --- | --- | --- | --- | --- |
| I Can Define The Basic Concepts Of Data Science. | Karaca et al. | Je peux définir les concepts de base de la science des données. | Data Science Basics | Ability to define basic concepts in AI and data science | Current AI Skills and Knowledge |
| I Can Define The Basic Concepts In Statistics. | Karaca et al. | Je peux définir les concepts de base en statistiques. | Statistics Basics | Ability to define basic concepts in AI and data science | Current AI Skills and Knowledge |
| I Can Explain How AI Systems Are Trained. | Karaca et al. | Je peux expliquer comment les systèmes d’IA sont entraînés. | AI Training Explained | Ability to define basic concepts in AI and data science | Current AI Skills and Knowledge |
| I Can Define The Basic Concepts And Terminology In AI. | Karaca et al. | Je peux définir les concepts et les terminologies de base en IA. | AI Concepts & Terminology | Ability to define basic concepts in AI and data science | Current AI Skills and Knowledge |
| I Can Accurately Analyze Data Obtained By AI In The Healthcare Field. | Karaca et al. | Je peux analyser correctement les données obtenues par l'IA dans le domaine de la santé. | AI Data Analysis in Health | Possession of analytical concepts in AI for health | Current AI Skills and Knowledge |
| I Can Differentiate Between The Functions And Features Of AI-Related Tools And Applications. | Karaca et al. | Je peux différencier les fonctions et les caractéristiques des outils et des applications liés à l'IA. | AI Tools & Features | Possession of analytical concepts in AI for health | Current AI Skills and Knowledge |
| I Can Articulate The Importance Of Data Collection, Analysis, Evaluation, And Security For The Development Of AI In Healthcare. | Karaca et al. | Je peux exprimer l'importance de la collecte, de l'analyse, de l'évaluation et de la sécurité des données pour le développement de l'IA dans la santé. | Data Importance in AI Health | Possession of analytical concepts in AI for health | Current AI Skills and Knowledge |
| I Can Access, Evaluate, Use, Share, And Create New Knowledge Using Information And Communication Technologies. | Karaca et al. | Je peux accéder, évaluer, utiliser, partager et créer de nouvelles connaissances en utilisant des technologies d’information et de communication. | ICT Knowledge Management | Possession of analytical concepts in AI for health | Current AI Skills and Knowledge |
| I Understand The Basic Computer Principles Of AI. | Sit et al. | Je comprends les principes informatiques de base de l'intelligence artificielle | AI Computing Principles | Understanding of AI techniques | Current AI Skills and Knowledge |
| I Am Comfortable With AI-Related Terminology. | Sit et al. | Je suis à l'aise avec la nomenclature relative à l'intelligence artificielle | AI Nomenclature | Understanding of AI techniques | Current AI Skills and Knowledge |
| I Understand How Computers Process Medical Imaging To Produce Visual Recognition And Analysis. | Li et al. | Je comprends comment les ordinateurs traitent l'imagerie médicale pour produire une reconnaissance et une analyse visuelles | Medical Imaging Processing | Understanding of AI techniques | Current AI Skills and Knowledge |
| I Understand Why AI-Assisted Genomic Diagnostics Require Massive Data ("Big Data") For Machine Learning. | Li et al. | Je comprends pourquoi les diagnostics génomiques assistés par l'IA ont besoin de données massives pour l’apprentissage automatique | Genomic AI & Big Data | Understanding of AI techniques | Current AI Skills and Knowledge |
| How Familiar Are You With AI | Boillat et al. | Dans quelle mesure êtes-vous familier avec l’intelligence artificielle | Familiarity with AI | Familiarity with general AI methods | Current AI Skills and Knowledge |
| How Familiar Are You With ML | Boillat et al. | Dans quelle mesure êtes-vous familier avec le machine learning | Familiarity with Machine Learning | Familiarity with general AI methods | Current AI Skills and Knowledge |
| How Familiar Are You With Supervised ML | Boillat et al. | Dans quelle mesure êtes-vous familier avec le machine learning supervisé | Familiarity with Supervised ML | Familiarity with general AI methods | Current AI Skills and Knowledge |
| How Familiar Are You With Unsupervised ML | Boillat et al. | Dans quelle mesure êtes-vous familier avec le machine learning non supervisé | Familiarity with Unsupervised ML | Familiarity with general AI methods | Current AI Skills and Knowledge |
| How Familiar Are You With Deep Learning | Boillat et al. | Dans quelle mesure êtes-vous familier avec le deep learning | Familiarity with Deep Learning | Familiarity with general AI methods | Current AI Skills and Knowledge |
| How Familiar Are You With Neural Networks | Boillat et al. | Dans quelle mesure êtes-vous familier avec les réseaux de neurones | Familiarity with Neural Networks | Familiarity with advanced AI techniques | Current AI Skills and Knowledge |
| How Familiar Are You With Fuzzy Logic | Boillat et al. | Dans quelle mesure êtes-vous familier avec le fuzzy logic | Familiarity with Fuzzy Logic | Familiarity with advanced AI techniques | Current AI Skills and Knowledge |
| How Familiar Are You With Support Vector Machines | Boillat et al. | Dans quelle mesure êtes-vous familier avec le support vector machine | Familiarity with Support Vector Machines | Familiarity with advanced AI techniques | Current AI Skills and Knowledge |
| How Familiar Are You With Overfitting/Underfitting | Boillat et al. | Dans quelle mesure êtes-vous familier avec le surapprentissage ou le sous-apprentissage | Familiarity with Overfitting/Underfitting | Familiarity with advanced AI techniques | Current AI Skills and Knowledge |
| How Familiar Are You With Feature Selection | Boillat et al. | Dans quelle mesure êtes-vous familier avec la sélection de paramètres | Familiarity with Feature Selection | Familiarity with advanced AI techniques | Current AI Skills and Knowledge |
| I Can Develop Work Plans That Are Compatible With AI | Karaca et al. | Je suis capable d'organiser des flux de travail compatibles avec l'intelligence artificielle | Organize AI-ready workflows | Ability to use AI for professional purposes | Practical Application and Clinical Integration of AI |
| I Can Leverage Information Derived From AI In Conjunction With My Professional Expertise | Karaca et al. | Je peux exploiter des informations issues de l’intelligence artificielle en les combinant avec mes connaissances professionnelles | Integrate AI insights | Ability to use AI for professional purposes | Practical Application and Clinical Integration of AI |
| I Can Use Artificial Intelligence Applications In Accordance With Their Intended Purpose | Karaca et al. | Je peux utiliser les logiciels d'intelligence artificielle conformément à leur objectif | Use AI software as intended | Ability to use AI for professional purposes | Practical Application and Clinical Integration of AI |
| I Can Use AI Technologies Effectively And Efficiently In The Delivery Of Care | Karaca et al. | Je peux utiliser les technologies de l'intelligence artificielle de manière efficace et efficiente dans la prestation de soins | Use AI effectively in care | Ability to use AI for clinical purposes | Practical Application and Clinical Integration of AI |
| I Can Explain How AI Applications Provide Solutions To Specific Healthcare Problems | Karaca et al. | Je peux expliquer comment les logiciels d'intelligence artificielle offrent une solution à tel ou tel problème dans la santé | Explain AI solutions in health | Ability to use AI for clinical purposes | Practical Application and Clinical Integration of AI |
| I Can Explain To Patients The AI Applications Used In Healthcare Services | Karaca et al. | Je peux expliquer au patient les logiciels d'intelligence artificielle utilisés dans les services de soin | Explain AI tools to patients | Ability to use AI for clinical purposes | Practical Application and Clinical Integration of AI |
| I Can Select The AI Application That Best Addresses The Problems Encountered In The Medical Field | Karaca et al. | Je peux choisir les logiciels d'intelligence artificielle correspondant aux problèmes rencontrés dans le domaine médical | Select AI tools for problems | Ability to use AI for clinical purposes | Practical Application and Clinical Integration of AI |
| By The End Of My Health Education, I Will Be Able To Confidently Use AI Tools In Healthcare When Needed | Sit et al. | À la fin de mon cursus en école de santé, je serai capable d'utiliser en confiance des outils d'intelligence artificielle dans le domaine de la santé, si nécessaire | Confidently use AI tools after my training | Expected ability, at the end of training, to work confidently with AI | Practical Application and Clinical Integration of AI |
| Overall, By The End Of My Medical Training, I Believe I Will Have The Necessary Knowledge To Work With AI In Routine Clinical Practice | Sit et al. | Dans l'ensemble, à la fin de mon cursus en école de santé, je pense que j’aurai les connaissances nécessaires pour travailler avec l'intelligence artificielle dans la pratique clinique courante | Able to work with AI after my training | Expected ability, at the end of training, to work confidently with AI | Practical Application and Clinical Integration of AI |
| I Am Convinced That I Will Succeed In Clinical Practice Involving Medical AI | Li et al. | Je suis persuadé que je réussirai dans la pratique clinique impliquant l'IA médicale | Confident to succeed in medical AI practice | Expected ability, at the end of training, to work confidently with AI | Practical Application and Clinical Integration of AI |
| I Will Pay Attention To Emerging AI Applications In Medical Practice | Li et al. | Je prêterai attention aux applications émergentes de l'IA utilisées dans la pratique médicale | Track emerging medical AI apps | Expected ability, at the end of training, to work confidently with AI | Practical Application and Clinical Integration of AI |
| Using Medical AI Enables Me To Accomplish My Clinical Tasks More Quickly | Li et al. | L’utilisation de l’IA médicale me permet d’accomplir mes tâches cliniques plus rapidement | AI speeds up my clinical tasks | Perceived impact of AI on individual productivity | Practical Application and Clinical Integration of AI |
| Using Medical AI Improves My Clinical Performance | Li et al. | L’utilisation de l’IA médicale améliore mes performances cliniques | AI improves my clinical performance | Perceived impact of AI on individual productivity | Practical Application and Clinical Integration of AI |
| Using Medical AI Increases My Clinical Productivity | Li et al. | L’utilisation de l'IA médicale augmente ma productivité clinique | AI boosts my clinical productivity | Perceived impact of AI on individual productivity | Practical Application and Clinical Integration of AI |
| Using Medical AI Improves My Efficiency | Li et al. | L'utilisation de l'IA médicale améliore mon efficacité | AI improves my efficiency | Perceived impact of AI on individual productivity | Practical Application and Clinical Integration of AI |
| I Understand How AI Optimizes Care Solutions. | Li et al. | Je comprends comment l’IA optimise les solutions de soins | Understand AI for care optimization | Understanding of how AI can optimize patient care | Practical Application and Clinical Integration of AI |
| I Understand How The AI Assistant In An Online Patient Triage System Manages Human–Machine Interaction | Li et al. | Je comprends comment l'assistant IA d’un système d'orientation des patients en ligne gère l'interaction homme-machine | Understand triage chatbot interaction | Understanding of how AI can optimize patient care | Practical Application and Clinical Integration of AI |
| I Believe That Using AI For Education, Service, And Research Is Beneficial | Karaca et al. | J'estime qu'il est utile d'utiliser l'intelligence artificielle à des fins d'éducation, de service et de recherche | AI useful for education, service and research | Expected impact that AI will have on the health system and professional careers | Attitudes and Perceptions Towards AI and Its Impact |
| AI Will Play An Important Role In Healthcare | Park et al. | L'intelligence artificielle jouera un rôle important dans le domaine de la santé | AI plays an important role in healthcare | Expected impact that AI will have on the health system and professional careers | Attitudes and Perceptions Towards AI and Its Impact |
| What Impact Do You Think Artificial Intelligence Will Have On The Practice Of Medicine Over The Course Of Your Medical Career? | Park et al. | L’IA aura un impact positif sur la pratique médicale au cours de ma carrière | AI will have a positive impact on medical practice | Expected impact that AI will have on the health system and professional careers | Attitudes and Perceptions Towards AI and Its Impact |
| I Am Less Likely To Consider A Career In Radiology, Given The Advancements In AI | Park et al. | Je suis MOINS susceptible d'envisager une carrière en radiologie, compte tenu des progrès de l'intelligence artificielle | Reduced interest in radiology careers due to AI | Expected impact that AI will have on the health system and professional careers | Attitudes and Perceptions Towards AI and Its Impact |
| Some Specialties Will Be Replaced By AI Within My Lifetime | Park et al. | Certaines spécialités seront remplacées par l'intelligence artificielle de mon vivant | AI will handle some specialties during my lifetime | Expected impact that AI will have on the health system and professional careers | Attitudes and Perceptions Towards AI and Its Impact |
| Teaching About Artificial Intelligence Will Be Beneficial For My Career | Sit et al. | L'enseignement de l'intelligence artificielle sera bénéfique pour ma carrière | AI education benefits my career | Perceived benefits of in-depth AI training | Training, Learning and Commitment to AI |
| All Health Students Should Receive Education On AI | Sit et al. | Tous les étudiants en faculté ou en école de santé devraient recevoir un enseignement sur l'intelligence artificielle | All health students should learn AI | Perceived benefits of in-depth AI training | Training, Learning and Commitment to AI |
| By The End Of My Health Education, I Will Have A Better Understanding Of The Methods Used To Evaluate The Performance Of AI Algorithms In Healthcare | Sit et al. | À la fin de mon cursus en santé, je comprendrai mieux les méthodes d'évaluation des performances des algorithmes d'intelligence artificielle en santé | Able to understand AI evaluation after my training | Perceived benefits of in-depth AI training | Training, Learning and Commitment to AI |
| Do You Think It Is Necessary To Receive More In-Depth Training In Order To Better Understand The Core Concept Of Ai | Boillat et al. | Pensez-vous nécessaire de bénéficier d'une formation plus approfondie pour mieux comprendre le concept principal de l’intelligence artificielle | Need more training for core AI concepts | Perceived need for in-depth AI training | Training, Learning and Commitment to AI |
| Do You Think It Is Necessary To Receive More In-Depth Training In Order To Explore New Opportunities In AI In General | Boillat et al. | Pensez-vous nécessaire de bénéficier d'une formation plus approfondie pour explorer les nouvelles possibilités de l'intelligence artificielle en général | Need more training to explore new AI possibilities | Perceived need for in-depth AI training | Training, Learning and Commitment to AI |
| Do You Think It Is Necessary To Receive More In-Depth Training In Order To Explore New Opportunities For AI In Healthcare And In My Field | Boillat et al. | Pensez-vous nécessaire de bénéficier d'une formation plus approfondie pour explorer les nouvelles opportunités de l'intelligence artificielle dans le domaine de la santé et dans mon domaine | Need more training to explore AI in healthcare/my field | Perceived need for in-depth AI training | Training, Learning and Commitment to AI |
| Do You Think It Is Necessary To Receive More In-Depth Training In Order To Learn More About Existing Commercial Solutions | Boillat et al. | Pensez-vous nécessaire de bénéficier d'une formation plus approfondie pour explorer les nouvelles opportunités de l'intelligence artificielle dans le domaine de la santé et dans mon domaine | Need more training to understand commercial AI solutions | Perceived need for in-depth AI training | Training, Learning and Commitment to AI |
| Do You Think It Is Necessary To Receive More In-Depth Training In Order To Develop My Own AI Algorithms Or Applications | Boillat et al. | Pensez-vous nécessaire de bénéficier d'une formation plus approfondie pour créer mes propres algorithmes ou applications d'intelligence artificielle | Need more training to build my own AI apps | Perceived need for in-depth AI training | Training, Learning and Commitment to AI |
| In Healthcare, Who Else Could Benefit From Additional Training And Educational Programs On Medical AI: Health Students | Boillat et al. | Dans le domaine de la santé, qui d'autre pourrait bénéficier de formations et de programmes éducatifs supplémentaires sur l’intelligence artificielle médicale : Étudiants en cursus de santé | Health students need AI education | Belief that all users, students and professionals, should be trained in AI | Training, Learning and Commitment to AI |
| In Healthcare, Who Else Could Benefit From Additional Training And Educational Programs On Medical AI: Medical Residents | Boillat et al. | Dans le domaine de la santé, qui d'autre pourrait bénéficier de formations et de programmes éducatifs supplémentaires sur l’intelligence artificielle médicale : Internes médicaux | Medical residents need AI education | Belief that all users, students and professionals, should be trained in AI | Training, Learning and Commitment to AI |
| In Healthcare, Who Else Could Benefit From Additional Training And Educational Programs On Medical AI: Practicing Health Professionals | Boillat et al. | Dans le domaine de la santé, qui d'autre pourrait bénéficier de formations et de programmes éducatifs supplémentaires sur l’intelligence artificielle médicale : Professionnels de santé en exercice | Practicing health professionals need AI education | Belief that all users, students and professionals, should be trained in AI | Training, Learning and Commitment to AI |
| In Healthcare, Who Else Could Benefit From Additional Training And Educational Programs On Medical AI: Public And Patients | Boillat et al. | Dans le domaine de la santé, qui d'autre pourrait bénéficier de formations et de programmes éducatifs supplémentaires sur l’intelligence artificielle médicale : Le grand public / les patients | Public and patients need AI education | Belief that all users, students and professionals, should be trained in AI | Training, Learning and Commitment to AI |
| In Healthcare, Who Else Could Benefit From Additional Training And Educational Programs On Medical AI: Hospital Administrators | Boillat et al. | Dans le domaine de la santé, qui d'autre pourrait bénéficier de formations et de programmes éducatifs supplémentaires sur l’intelligence artificielle médicale : Administrateurs d'hôpitaux | Hospital administrators need AI education | Belief that all users, students and professionals, should be trained in AI | Training, Learning and Commitment to AI |
| In Healthcare, Who Else Could Benefit From Additional Training And Educational Programs On Medical AI : Policy Makers | Boillat et al. | Dans le domaine de la santé, qui d'autre pourrait bénéficier de formations et de programmes éducatifs supplémentaires sur l’intelligence artificielle médicale : Décideurs politiques | Policy makers need AI education | Belief that all users, students and professionals, should be trained in AI | Training, Learning and Commitment to AI |
| In Healthcare, Who Else Could Benefit From Additional Training And Educational Programs On Medical AI : Nursing Staff | Boillat et al. | Dans le domaine de la santé, qui d'autre pourrait bénéficier de formations et de programmes éducatifs supplémentaires sur l’intelligence artificielle médicale : Le personnel soignant | Nursing staff need AI education | Belief that all users, students and professionals, should be trained in AI | Training, Learning and Commitment to AI |
| My School Organizes Advanced Courses To Help Us Learn More About Medical AI Technologies. | Li et al. | Mon école organise des cours d'approfondissement pour nous permettre d'en savoir plus sur les technologies d'IA médicale | My school offers advanced medical AI courses | Perceived encouragement from peers and entourage to use AI in health | Training, Learning and Commitment to AI |
| My Peers And/Or Parents Encourage Me To Participate In Innovative Activities To Learn About Medical AI | Li et al. | Mes pairs et/ou mes parents m'encouragent à participer à des activités innovantes d'apprentissage de l'IA médicale | Peers/parents encourage innovative AI learning | Perceived encouragement from peers and entourage to use AI in health | Training, Learning and Commitment to AI |
| My Supervisors Have Emphasized The Need To Work Creatively Using Medical AI | Li et al. | Mes encadrants / supérieurs ont insisté sur la nécessité de travailler de manière créative à l’aide de l'IA médicale | Supervisors stress creative work with medical AI | Perceived encouragement from peers and entourage to use AI in health | Training, Learning and Commitment to AI |
| My Peers Believe That It Is Necessary To Learn How To Work With Medical AI | Li et al. | Mes pairs estiment qu'il est nécessaire d'apprendre à travailler avec l’IA médicale. | Peers say learning medical AI is necessary | Perceived encouragement from peers and entourage to use AI in health | Training, Learning and Commitment to AI |
| I Am Confident That I Can Understand The Most Challenging Materials Presented In Medical AI Courses | Li et al. | Je suis certain de pouvoir comprendre les documents les plus difficiles présentés dans les cours sur l'IA médicale | Confident to understand toughest course materials | Confidence in the ability to learn how to use AI | Training, Learning and Commitment to AI |
| I Am Confident That I Can Learn The Basic Concepts Taught In Medical AI Courses | Li et al. | Je suis certain de pouvoir apprendre les concepts de base enseignés dans les cours sur l'IA médicale | Confident to learn basic AI course concepts | Confidence in the ability to learn how to use AI | Training, Learning and Commitment to AI |
| I Will Continue To Educate Myself About Medical AI Technology | Li et al. | Je continuerai à me renseigner sur la technologie de l'IA médicale | I will keep learning about medical AI technology | Commitment to learning about AI | Training, Learning and Commitment to AI |
| I Believe I Will Keep Track Of The Future Development Of Medical AI | Li et al. | Je pense que je prêterai attention au développement de l'IA médicale à l'avenir | I will follow medical AI development | Commitment to learning about AI | Training, Learning and Commitment to AI |
| I Intend To Dedicate Time To Learning About Medical AI | Li et al. | J'ai l'intention de consacrer du temps à l'apprentissage de l'IA médicale | I intend to spend time learning medical AI | Commitment to learning about AI | Training, Learning and Commitment to AI |
| I Have Actively Sought Out And Watched Educational Videos About Medical AI | Li et al. | J'ai intentionnellement cherché et visionné des vidéos pédagogiques concernant l'IA médicale | I have watched medical AI educational videos | Previous knowledge and exposure to AI | Training, Learning and Commitment to AI |
| I Have Studied Medical AI Applications To Understand How They Work | Li et al. | J'ai étudié des applications médicales d'IA pour comprendre leur fonctionnement | I have studied medical AI apps | Previous knowledge and exposure to AI | Training, Learning and Commitment to AI |
| I Have Studied Medical AI In Books And Journals | Li et al. | J'ai étudié l'IA médicale dans des livres et des revues | I have studied medical AI in books/journals | Previous knowledge and exposure to AI | Training, Learning and Commitment to AI |
| I Have Attended Courses On Medical AI Either At My Institution Or Externally. | Li et al. | J'ai assisté à des cours sur l'IA médicale dans des écoles ou à l’extérieur | I have attended medical AI courses | Previous knowledge and exposure to AI | Training, Learning and Commitment to AI |
| I Can Explain The Limitations Of AI Technologies | Karaca et al. | Je peux expliquer les limites des technologies de l'intelligence artificielle | Explain AI limitations | AI strengths, limits and risks | Ethical, Regulatory and Risk Aspects of AI |
| I Can Explain The Strengths And Weaknesses Of AI Technologies | Karaca et al. | Je peux expliquer les forces et les faiblesses des technologies de l’intelligence artificielle | Explain AI strengths and weaknesses | AI strengths, limits and risks | Ethical, Regulatory and Risk Aspects of AI |
| I Can Consider The Opportunities And Threats That AI Technologies May Pose | Karaca et al. | Je peux envisager les opportunités et les menaces que les technologies de l’intelligence artificielle peuvent engendrer | Assess AI opportunities and threats | AI strengths, limits and risks | Ethical, Regulatory and Risk Aspects of AI |
| I Understand The Limitations Of Artificial Intelligence | Sit et al. | Je comprends les limites de l'intelligence artificielle | Understand AI limitations | AI strengths, limits and risks | Ethical, Regulatory and Risk Aspects of AI |
| I Can Use Health-Related Data In Compliance With Legal And Ethical Standards | Karaca et al. | Je peux utiliser les données relatives à la santé dans le respect des normes juridiques et éthiques | Use health data within legal and ethical norms | Legal and ethical use of AI | Ethical, Regulatory and Risk Aspects of AI |
| I Can Adhere To Ethical Principles When Using AI | Karaca et al. | Je peux suivre des principes éthiques lorsque j'utilise l’intelligence artificielle | Follow ethical principles when using AI | Legal and ethical use of AI | Ethical, Regulatory and Risk Aspects of AI |
| I Can Follow The Legal Regulations Regarding The Use Of AI In Healthcare | Karaca et al. | Je peux suivre les réglementations légales concernant l'utilisation de l'intelligence artificielle dans le domaine de la santé | Follow legal regulations for AI in healthcare | Legal and ethical use of AI | Ethical, Regulatory and Risk Aspects of AI |
| In Your Field, Which Of The Following Issues Are Important For The Development And Implementation Of Medical AI: Outcomes Of AI Algorithms Are Difficult To Trace Or Understand (The Blackbox Syndrome) | Boillat et al. | Dans votre domaine, quels sont les sujets importants pour le développement et la mise en œuvre de l’intelligence artificielle médicale parmi celles ci-dessous : Les résultats des algorithmes d'intelligence artificielle sont difficiles à retracer ou à comprendre | Black-box traceability | Implementation challenges in health AI | Ethical, Regulatory and Risk Aspects of AI |
| In Your Field, Which Of The Following Issues Are Important For The Development And Implementation Of Medical Ai: The Complexity Of The Field Of Medicine | Boillat et al. | Dans votre domaine, quels sont les sujets importants pour le développement et la mise en œuvre de l’intelligence artificielle médicale parmi celles ci-dessous : La complexité du domaine médical | Clinical complexity | Implementation challenges in health AI | Ethical, Regulatory and Risk Aspects of AI |
| In Your Field, Which Of The Following Issues Are Important For The Development And Implementation Of Medical Ai: The Availability Of High Quality Data Samples | Boillat et al. | Dans votre domaine, quels sont les sujets importants pour le développement et la mise en œuvre de l’intelligence artificielle médicale parmi celles ci-dessous : La disponibilité d'échantillons de données de haute qualité | High-quality data availability | Implementation challenges in health AI | Ethical, Regulatory and Risk Aspects of AI |
| In Your Field, Which Of The Following Issues Are Important For The Development And Implementation Of Medical AI: The AI's Level Of Autonomy (What AI Should And Should Not Do) | Boillat et al. | Dans votre domaine, quels sont les sujets importants pour le développement et la mise en œuvre de l’intelligence artificielle médicale parmi celles ci-dessous : Le niveau d'autonomie de l'intelligence artificielle | Appropriate autonomy level | Implementation challenges in health AI | Ethical, Regulatory and Risk Aspects of AI |
| In Your Field, Which Of The Following Issues Are Important For The Development And Implementation Of Medical Ai: The Costs Associated With The Implementation Of AI | Boillat et al. | Dans votre domaine, quels sont les sujets importants pour le développement et la mise en œuvre de l’intelligence artificielle médicale parmi celles ci-dessous : Les coûts associés à la mise en œuvre de l'intelligence artificielle | Implementation costs | Implementation challenges in health AI | Ethical, Regulatory and Risk Aspects of AI |
| In Your Field, Which Of The Following Issues Are Important For The Development And Implementation Of Medical AI: Data Privacy / Confidentiality | Boillat et al. | Dans votre domaine, quels sont les sujets importants pour le développement et la mise en œuvre de l’intelligence artificielle médicale parmi celles ci-dessous : La confidentialité des données | Data privacy | Implementation challenges in health AI | Ethical, Regulatory and Risk Aspects of AI |
| In Your Field, Which Factors Are The Most Important For Driving The Implementation Of Medical Ai: The Availability Of Comparison Studies | Boillat et al. | Dans votre domaine, quels sont les facteurs les plus importants pour la mise en œuvre de l'intelligence artificielle médicale : La disponibilité d'études comparatives | Comparative evidence | Evidence and safety requirements | Ethical, Regulatory and Risk Aspects of AI |
| In Your Field, Which Factors Are The Most Important For Driving The Implementation Of Medical AI: The Safe Use Of AI | Boillat et al. | Dans votre domaine, quels sont les facteurs les plus importants pour la mise en œuvre de l'intelligence artificielle médicale : L'utilisation de l'intelligence artificielle en toute sécurité | Safe use of AI | Evidence and safety requirements | Ethical, Regulatory and Risk Aspects of AI |
| In Your Field, Which Factors Are The Most Important For Driving The Implementation Of Medical AI: Build Trust Between Humans And AI | Boillat et al. | Dans votre domaine, quels sont les facteurs les plus importants pour la mise en œuvre de l'intelligence artificielle médicale : Instaurer la confiance entre les humains et l'intelligence artificielle | Human-AI trust | Evidence and safety requirements | Ethical, Regulatory and Risk Aspects of AI |
| In Your Field, Which Factors Are The Most Important For Driving The Implementation Of Medical AI: Availability Of Regulations And Legislations | Boillat et al. | Dans votre domaine, quels sont les facteurs les plus importants pour la mise en œuvre de l'intelligence artificielle médicale :La disponibilité des règlements et des législations | Regulatory readiness | Evidence and safety requirements | Ethical, Regulatory and Risk Aspects of AI |
| In Your Field, Which Factors Are The Most Important For Driving The Implementation Of Medical AI: The Top Management's Level Of Understanding | Boillat et al. | Dans votre domaine, quels sont les facteurs les plus importants pour la mise en œuvre de l'intelligence artificielle médicale : Le niveau de compréhension de la haute direction | Leadership understanding | Evidence and safety requirements | Ethical, Regulatory and Risk Aspects of AI |
| What Do You Consider The Risks Of Medical AI: Dehumanization Of Healthcare | Boillat et al. | Selon vous, quels sont les risques de l'intelligence artificielle médicale : La déshumanisation des services de soins | Dehumanization of care | Perceived risks of health AI | Ethical, Regulatory and Risk Aspects of AI |
| What Do You Consider The Risks Of Medical AI: Reduction In Physicians' Skills (E.G., Physicians Might Execute Fewer Types Of Tasks) | Boillat et al. | Selon vous, quels sont les risques de l'intelligence artificielle médicale : La baisse des compétences des professionnels de santé | Clinician skill erosion | Perceived risks of health AI | Ethical, Regulatory and Risk Aspects of AI |
| What Do You Consider The Risks Of Medical AI: AI Will Eventually Harm Patients | Boillat et al. | Selon vous, quels sont les risques de l'intelligence artificielle médicale : L’intelligence artificielle finira par nuire aux patients | Patient harm | Perceived risks of health AI | Ethical, Regulatory and Risk Aspects of AI |
| What Do You Consider The Risks Of Medical AI: Physicians May Become Redundant | Boillat et al. | Selon vous, quels sont les risques de l'intelligence artificielle médicale : Les praticiens peuvent devenir superflus | Clinician displacement | Perceived risks of health AI | Ethical, Regulatory and Risk Aspects of AI |

Karaca O, Çalışkan SA, Demir K. Medical artificial intelligence readiness scale for medical students (MAIRS-MS)-development, validity and reliability study. BMC Med Educ 2021 Dec;21(1):112. doi: 10.1186/s12909-021-02546-6

Sit C, Srinivasan R, Amlani A, Muthuswamy K, Azam A, Monzon L, Poon DS. Attitudes and perceptions of UK medical students towards artificial intelligence and radiology: a multicentre survey. Insights Imaging 2020 Dec;11(1):14. doi: 10.1186/s13244-019-0830-7

Li X, Jiang MY, Jong MS, Zhang X, Chai C. Understanding Medical Students’ Perceptions of and Behavioral Intentions toward Learning Artificial Intelligence: A Survey Study. IJERPH 2022 July 18;19(14):8733. doi: 10.3390/ijerph19148733

Boillat T, Nawaz FA, Rivas H. Readiness to Embrace Artificial Intelligence Among Medical Doctors and Students: Questionnaire-Based Study. JMIR Med Educ 2022 Apr 12;8(2):e34973. doi: 10.2196/34973

Park CJ, Yi PH, Siegel EL. Medical Student Perspectives on the Impact of Artificial Intelligence on the Practice of Medicine. Current Problems in Diagnostic Radiology 2021 Sept;50(5):614–619. doi: 10.1067/j.cpradiol.2020.06.011

**Table S3. Post hoc descriptive thematic summaries: theme scores and internal coherence indicators**

Theme scores are arithmetic means of the constituent Likert-type items, each scored from 1 to 5, with higher values indicating stronger agreement or endorsement. Confidence intervals for theme scores were recalculated using participant-level theme scores (mean of each participant's responses to the theme's constituent items; n=190). Cronbach α is reported as an internal coherence indicator for descriptive interpretation only and is not intended as evidence of formal scale validation.

| Aggregated Domain | Specific Theme | Descriptive Theme Score, Mean (95% CI) | Cronbach α | Items, n | Internal Coherence Comment |
| --- | --- | --- | --- | --- | --- |
| Current AI Skills And Knowledge | Ability to define basic concepts in AI and data science | 2.58  (2.47-2.69) | 0.71 | 4 | **Overall internal coherence acceptable**  two themes slightly below threshold (α =0.68–0.69), retained |
|  | Possession of analytical concepts in AI for health | 2.89 (2.78-3.00) | 0.69^a^ | 4 |  |
|  | Understanding of AI techniques | 2.54 (2.43-2.65) | 0.68^a^ | 4 |  |
|  | Familiarity with general AI methods | 1.82 (1.71-1.93) | 0.80 | 5 |  |
|  | Familiarity with advanced AI techniques | 1.20 (1.13-1.27) | 0.70 | 5 |  |
| Practical Application and Clinical Integration Of AI | Ability to use AI for professional purposes | 3.13 (3.01-3.25) | 0.69^a^ | 3 | **Internal coherence acceptable to high**  one theme slightly below the conventional α threshold (α=0.69) |
|  | Ability to use AI for clinical purposes | 2.05 (1.93-2.16) | 0.79 | 4 |  |
|  | Expected ability, at the end of training, to work confidently with AI | 2.63  (2.53-2.73) | 0.85 | 4 |  |
|  | Perceived impact of AI on individual productivity | 1.99  (1.84-2.14) | 0.95 | 4 |  |
|  | Understanding of how AI can optimize patient care | 2.02 (1.89-2.15) | 0.77 | 2 |  |
| Attitudes And Perceptions Towards AI And Its Impact | Expected impact that AI will have on the health system and professional careers | 3.50^b^ | 0.48^a^ | 5 | Considered as one block due to conceptual diversity despite low α (0.48) |
| Training, Learning And Commitment To AI | Perceived benefits of in-depth AI training | 3.43^b^  (3.34-3.52) | 0.47^a^ | 3 | **Overall internal coherence acceptable**  two themes with lower α (0.47 and 0.58) retained after content coherence review |
|  | Perceived need for in-depth AI training | 3.69 (3.59-3.79) | 0.79 | 5 |  |
|  | Belief that all users, students and professionals, should be trained in AI | 4.11 (4.02-4.20) | 0.90 | 7 |  |
|  | Perceived encouragement from peers and entourage to use AI in health | 1.43  (1.34-1.52) | 0.82 | 4 |  |
|  | Confidence in the ability to learn how to use AI | 2.47^b^  (2.35-2.5) | 0.58^a^ | 2 |  |
|  | Commitment to learning about AI | 2.76  (2.63-2.89) | 0.86 | 3 |  |
|  | Previous knowledge and exposure to AI | 1.36 (1.27-1.45) | 0.85 | 4 |  |
| Ethical, Regulatory And Risk Aspects Of AI | AI strengths, limits and risks | 3.42 (3.31-3.53) | 0.82 | 4 | **Overall internal coherence acceptable**  one theme with lower α (0.61)  retained after content coherence review |
|  | Legal and ethical use of AI | 3.25  (3.15-3.35) | 0.61^a^ | 3 |  |
|  | Implementation challenges in health AI | 3.89 (3.81-3.97) | 0.76 | 6 |  |
|  | Evidence and safety requirements | 4.04  (3.96-4.12) | 0.75 | 5 |  |
|  | Perceived risks of health AI | 3.60 (3.48-3.72) | 0.73 | 4 |  |

**^a^** α < 0.70; retained for descriptive thematic completeness after review of item content and internal coherence

ᵇ Composite reported descriptively because of heterogeneous items and/or low internal consistency (α < 0.60); the mean should not be interpreted as a unidimensional scale score and was not used for inferential comparisons

**Table S4. Descriptive contextualization matrix: selected survey patterns and illustrative exploratory interview observations**

This table is provided as a descriptive contextualization matrix. It links selected survey patterns with illustrative exploratory interview observations from the single-site qualitative follow-up strand. The qualitative observations are not intended to explain, confirm, or generalize the quantitative findings, and no mixed-methods inference is claimed. Theme scores are reported descriptively; full item-level results and post hoc thematic summaries are provided in Tables S2 and S3.

| Aggregated Domain | Salient Survey Pattern | Illustrative Interview Observation (English Translation) | Descriptive Contextual Note |
| --- | --- | --- | --- |
| Current AI Skills And Knowledge | Very low reported technical familiarity (eg, advanced AI techniques theme mean 1.20; high disagreement for supervised ML items)  Foundational knowledge was uneven (eg, statistics basics item showed higher agreement than algorithm-specific items) | “Je ne saurais pas exactement bien définir la notion d’IA.” *(I would not really be able to define AI.)*  “Je connais les IA génératives : ChatGPT…” *(I mainly know generative AI tools such as ChatGPT.)* | Students’ self-reported AI knowledge appeared largely conceptual and non-technical. The interview observations illustrate that AI was often discussed through everyday generative AI tools and broad narratives rather than through specific machine-learning concepts.  This may help contextualize the contrast between general AI familiarity and low familiarity with specific AI methods. |
| Practical Application And Clinical Integration | Low perceived readiness for clinical use (eg, clinical use theme mean 2.56; high disagreement for selecting tools and explaining AI to patients)  Professional use outside patient care was closer to neutral | “Aucun usage de l’IA en stage pratique.” *(No AI use during placements.)*  “En stage j’utilise ChatGPT… quand j’ai besoin d’une réponse rapide.” *(During placements I use ChatGPT when I need a quick answer.)* | AI was not commonly described as embedded in supervised placement routines. The interview observations illustrate limited exposure to structured practice, feedback, or explicit discussion of AI-supported workflows. When AI use was mentioned, it generally referred to unsupervised use of general-purpose generative AI tools, highlighting the relevance of guidance on verification, boundaries, and safe use. |
| Attitudes And Perceptions Towards AI | Overall views were above neutral (eg, most students agreed AI will play an important role in healthcare and have a positive career impact), but concerns about relational care and dehumanization were salient. | “L’IA va avoir une place importante… mais pas remplacer l’humain.” *(AI will have an important role, but not replace humans.)*  “Il est important de garder la clinique, parler à la personne.” *(It is important to keep the clinical and relational dimension.)* | Students’ acceptance of AI appeared conditional. The interview observations illustrate that AI was viewed more favorably when framed as supporting, rather than replacing, human judgment and relational care.  This contextualizes the importance of connecting AI education with shared decision-making, professional responsibility, and patient-centered communication in midwifery. |
| Training, Learning And Commitment To AI | Strong endorsement of AI education (eg, belief that students/professionals should be trained: theme mean ~4.11) contrasted with very low perceived institutional/social support and limited prior exposure (eg, prior exposure ~1.36; encouragement ~1.43). | “Je n’ai pas vraiment eu de formation.” *(I have not really had any training.)*  “J’aimerais qu’on nous explique comment utiliser ces outils.” *(I would like someone to explain how to use these tools.)* | The survey pattern illustrates a contrast between strong perceived need for AI education and low prior exposure or perceived support. The interview observations are consistent with this gap and suggest that students may currently rely on informal or heterogeneous learning opportunities.  This points to the relevance of explicit learning outcomes, curricular integration, and supervised opportunities to discuss AI use in practice. |
| Ethical, Regulatory And Risk Aspects Of AI | Ethics and governance themes were above neutral, with the highest scores for evidence and safety requirements (eg, theme mean 4.04) and high salience of data privacy and traceability issues. | “Il faudrait que ce soit vraiment fiable concernant la sécurité des données.” *(It must be truly reliable regarding data security.)*  “C’est important de toujours vérifier.” *(It is important to always verify.)* | Safety, accountability, verification, and data protection were salient across the survey findings. The interview observations illustrate similar concerns while suggesting limited familiarity with concrete regulatory or governance mechanisms.  These patterns suggest that governance-oriented teaching could use students’ ethical sensitivity as an entry point for addressing regulatory literacy, validation principles, and accountability in practice-based scenarios. |
| Cross-Cutting: AI Label Ambiguity / GenAI Salience | Item-level responses suggest a specific inconsistency: endorsement of “Familiarity with AI” appears higher than endorsement of “Familiarity with ML / supervised ML / unsupervised ML / deep learning / neural networks,” which cluster in strong disagreement. | “Je connais les IA génératives : ChatGPT…” *(I mainly know generative AI tools such as ChatGPT.)*  “Ça a surtout commencé avec ChatGPT…” *(It mostly started with ChatGPT…)* | The contrast between higher self-reported familiarity with “AI” and lower familiarity with machine-learning methods raises the hypothesis that some students may interpret AI primarily through consumer-facing generative AI tools. The interview observations, including references to ChatGPT, are consistent with this hypothesis.  This reinforces the relevance of distinguishing general AI, generative AI tools, and regulated clinical AI systems in future measurement and curriculum design. |

The quantitative instruments did not include items specific to generative AI. Exploratory interviews illustrated that some students referred to AI primarily through general-purpose generative tools, raising the possibility of conflation with regulated clinical AI systems. This observation may inform future measurement and curriculum design.
