# Supplementary material for: AI Literacy and Training Needs Among French Midwifery Students: National Cross-Sectional Online Survey and Exploratory Qualitative Follow-Up
Source: JMIR Med Educ. 2026 Oct 26;12:e94310. doi: 10.2196/94310 (PMC13706253; doi:10.2196/94310)
Supplement: Supplementary file 2 [file mededu-v12-e94310-s002.docx]

# STROBE Checklist for Cross-Sectional Study

Study title: AI Literacy and Training Needs Among French Midwifery Students: National Cross-Sectional Online Survey and Exploratory Qualitative Follow-Up.

von Elm E, Altman DG, Egger M, Pocock SJ, Gøtzsche PC, Vandenbroucke JP; STROBE Initiative. The Strengthening the Reporting of Observational Studies in Epidemiology (STROBE) statement: guidelines for reporting observational studies. Lancet. 2007 Oct 20;370(9596):1453-7. doi: 10.1016/S0140-6736(07)61602-X. PMID: 18064739.

Note: The STROBE checklist was applied to the quantitative cross-sectional survey component of this study.

| **Section** | **Item** | **STROBE Recommendation** | **Reported on page/section** | **Done** |
| --- | --- | --- | --- | --- |
| Title and abstract | 1 | Indicate the study design with a commonly used term in the title or abstract. | Title, Abstract | Yes |
| Introduction | 2 | Explain the scientific background and rationale for the investigation. | Introduction | Yes |
| Introduction | 3 | State specific objectives, including any prespecified hypotheses. | Introduction, last paragraph | Yes |
| Methods | 4 | Present key elements of study design early in the paper. | Methods, “Study Design” | Yes |
| Methods | 5 | Describe the setting, locations, and relevant dates, including periods of recruitment and data collection. | Methods, “Setting, Population and Recruitment”; “Survey Administration” | Yes |
| Methods | 6 | Give the eligibility criteria and the sources and methods of selection of participants. | Methods, “Setting, Population and Recruitment” | Yes |
| Methods | 7 | Clearly define all variables, outcomes, exposures, predictors, potential confounders, and effect modifiers. | Methods, “Survey Instruments”; “Item Aggregation and Thematic Domain Mapping” | Yes |
| Methods | 8 | For each variable of interest, give sources of data and details of methods of assessment. | Methods, “Survey Instruments”; “Data Management and Statistical Analysis” | Yes |
| Methods | 9 | Describe any efforts to address potential sources of bias. | Methods, “Survey Administration”; Discussion, “Strengths and Limitations” | Yes |
| Methods | 10 | Explain how the study size was arrived at. | Methods, “Setting, Population and Recruitment”; “Data Management and Statistical Analysis” | N/A: no formal sample-size or power calculation was performed; the study used voluntary convenience sampling. |
| Methods | 11 | Explain how quantitative variables were handled in the analyses. | Methods, “Data Management and Statistical Analysis”; “Item Aggregation and Thematic Domain Mapping” | Yes |
| Methods | 12 | Describe all statistical methods, including those used to control for confounding. | Methods, “Data Management and Statistical Analysis” | Partial: no methods to control for confounding were used |
| Results | 13 | Report numbers of individuals at each stage of the study. | Results, “Participation and Respondent Characteristics” | Yes |
| Results | 14 | Give characteristics of study participants. | Results, Table 1 | Yes |
| Results | 15 | Report numbers of outcome events or summary measures. | Results; Figures 1–5 | Yes |
| Results | 16 | Give unadjusted and, if applicable, adjusted estimates with confidence intervals. | Results; Figures 1–5; Multimedia Appendix 1, Table S3 | Yes: descriptive, unadjusted estimates with 95% CIs at the theme-score level; no adjusted estimates were produced |
| Results | 17 | Report other analyses, such as subgroup, interaction, or sensitivity analyses. | Not applicable | N/A: no subgroup, interaction, or sensitivity analyses were conducted. |
| Discussion | 18 | Summarize key results with reference to study objectives. | Discussion, “Principal Findings” | Yes |
| Discussion | 19 | Discuss limitations of the study, considering sources of potential bias or imprecision. | Discussion, “Strengths and Limitations” | Yes |
| Discussion | 20 | Give a cautious overall interpretation considering objectives, limitations, multiplicity of analyses, results from similar studies, and other evidence. | Discussion, “Interpretation and Comparison With Prior Work”; “Strengths and Limitations” | Yes |
| Discussion | 21 | Discuss the generalisability or external validity of the study results. | Discussion, “Strengths and Limitations”; Conclusions | Yes |
| Other information | 22 | Give the source of funding and the role of the funders. | Funding statement / Acknowledgments | Yes |

# COREQ Consolidated Criteria for Reporting Qualitative Research Checklist

Study title: AI Literacy and Training Needs Among French Midwifery Students: National Cross-Sectional Online Survey and Exploratory Qualitative Follow-Up.

Tong A, Sainsbury P, Craig J. Consolidated criteria for reporting qualitative research (COREQ): a 32-item checklist for interviews and focus groups. Int J Qual Health Care. 2007;19(6):349-357.

Note: This COREQ checklist applies to the qualitative component (semi-structured interviews).

**Domain 1: Research team and reflexivity**

| **Item** | **Question / Description** | **Reported in manuscript (section)** | **Done** |
| --- | --- | --- | --- |
| 1 | Interviewer/facilitator: Which author(s) conducted the interviews or focus groups? | Methods, “Qualitative Follow-Up Strand”: interviews were conducted by a member of the research team who was a midwife. | Partial: interviewer role reported; specific author not explicitly named in the Methods. |
| 2 | Credentials: What were the researcher’s credentials? | Methods, “Qualitative Follow-Up Strand”: interviewer reported as a midwife. | Partial: “midwife” reported; academic credentials not otherwise specified. |
| 3 | Occupation: What was their occupation at the time of the study? | Methods, “Qualitative Follow-Up Strand”: member of the research team and midwife. | Partial: role as midwife/research team member reported; detailed occupation not further specified. |
| 4 | Gender: Was the researcher male or female? | Not reported. | Not reported. |
| 5 | Experience and training: What experience or training did the researcher have? | Not reported. | Not reported. |
| 6 | Relationship established: Was a relationship established prior to study commencement? | Not reported. | Not reported. |
| 7 | Participant knowledge of the interviewer: What did participants know about the researcher? | Ethical information given to participants is reported, but participant knowledge of the interviewer is not described. | Not reported. |
| 8 | Interviewer characteristics: What characteristics were reported about the interviewer/facilitator, such as biases, assumptions, reasons, or interests in the research topic? | Methods, “Qualitative Follow-Up Strand”: analytic memos and reflexive notes were maintained. | Partial: reflexive documentation reported; interviewer assumptions, biases, or reasons for interest are not explicitly described. |

**Domain 2: Study design**

| **Item** | **Question / Description** | **Reported in manuscript (section)** | **Done** |
| --- | --- | --- | --- |
| 9 | Methodological orientation and theory: What methodological orientation was stated? | Methods, “Qualitative Follow-Up Strand”: pragmatic reflexive thematic approach informed by Braun and Clarke. | Yes. |
| 10 | Sampling: How were participants selected? | Methods, “Qualitative Follow-Up Strand”: volunteer midwifery students from one school in eastern France; attention to variation in year of study, gender, and prior exposure to AI or digital health. | Yes: volunteer, single-site, pragmatic sampling with attention to variation. |
| 11 | Method of approach: How were participants approached? | Methods, “Qualitative Follow-Up Strand”: students were invited during teaching sessions and by email through a local coordinator. | Yes. |
| 12 | Sample size: How many participants were in the study? | Methods, “Qualitative Follow-Up Strand”; Results, “Participation and Respondent Characteristics”: eight students were interviewed. | Yes. |
| 13 | Non-participation: How many people refused to participate or dropped out? | Not reported. | Not reported. |
| 14 | Setting of data collection: Where was the data collected? | Methods, “Qualitative Follow-Up Strand”: interviews took place either face-to-face in a quiet room at the school or via secure videoconference. | Yes. |
| 15 | Presence of non-participants: Was anyone else present besides participants and researchers? | Not reported. | Not reported. |
| 16 | Description of sample: What are the important characteristics of the sample? | Results, “Participation and Respondent Characteristics”; Table 1: eight volunteer students, years 3–5, no male participants, no second-year students; age and year of study reported. | Yes. |
| 17 | Interview guide: Were questions, prompts, or guides provided? Was it pilot-tested? | Methods, “Qualitative Follow-Up Strand”: an interview guide was used, informed by the literature, the five questionnaires, and preliminary survey findings; it was not formally pilot-tested. | Yes: guide used; not formally pilot-tested. |
| 18 | Repeat interviews: Were repeat interviews carried out? | No repeat interviews are described. | Not reported. |
| 19 | Audio/visual recording: Did the research use audio or visual recording? | Methods, “Qualitative Follow-Up Strand”: interviews were audio-recorded with consent. | Yes. |
| 20 | Field notes: Were field notes made during and/or after the interview? | Methods, “Qualitative Follow-Up Strand”: analytic memos and reflexive notes were maintained. | Partial: analytic/reflexive notes reported; timing relative to interviews not specified. |
| 21 | Duration: What was the duration of each interview? | Methods, “Qualitative Follow-Up Strand”: interviews lasted a maximum of 30 minutes. | Yes. |
| 22 | Data saturation: Was data saturation discussed? | Methods, “Study Design”; Discussion, “Strengths and Limitations”: no claim of data saturation is made because the qualitative strand was exploratory, single-site, and small-sample. | No: saturation not claimed; no formal saturation procedure. |
| 23 | Transcripts returned: Were transcripts returned to participants for comment or correction? | Methods, “Qualitative Follow-Up Strand”: member checking was not conducted. | No: transcripts were not reported as returned; member checking was not conducted. |

**Domain 3: Analysis and findings**

| **Item** | **Question / Description** | **Reported in manuscript (section)** | **Done** |
| --- | --- | --- | --- |
| 24 | Number of data coders: How many researchers coded the data? | Methods, “Qualitative Follow-Up Strand”: descriptive codes were assigned by SKn; SKo independently reviewed the coding framework and thematic structure. | Yes: one primary coder and one independent reviewer. |
| 25 | Description of the coding tree: Did authors provide a description of the coding tree? | Methods, “Qualitative Follow-Up Strand”: coding framework and thematic structure reviewed; discrepancies discussed; codebook refined iteratively. | Partial: coding process and codebook refinement described; detailed coding tree not provided. |
| 26 | Derivation of themes: Were themes identified in advance or derived from the data? | Methods, “Qualitative Follow-Up Strand”: analysis involved familiarization, initial coding, grouping of related codes, theme development and refinement, and review against the study questions. | Yes: themes were developed through thematic analysis and reviewed against the study questions. |
| 27 | Software: What software, if any, was used to manage the data? | Methods, “Qualitative Follow-Up Strand”: coding was managed manually in a structured spreadsheet rather than dedicated qualitative-analysis software. | Yes. |
| 28 | Participant checking: Did participants provide feedback on the findings? | Methods, “Qualitative Follow-Up Strand”: member checking was not conducted. | No. |
| 29 | Quotations presented: Were participant quotations presented to illustrate the themes or findings? | Multimedia Appendix 1, Table S4: illustrative interview observations/quotes are presented to contextualize selected survey patterns. | Yes: Illustrative interview observations/quotes are presented in Multimedia Appendix 1. |
| 30 | Data and findings consistent: Was there consistency between the data presented and the findings? | Results sections and Multimedia Appendix 1, Table S4: qualitative observations are presented as exploratory contextual illustrations, not as independent confirmation. | Yes: qualitative observations are presented cautiously and consistently with the exploratory framing. |
| 31 | Clarity of major themes: Were major themes clearly presented? | Results sections and Multimedia Appendix 1, Table S4: qualitative observations are organized around the five survey domains. | Partial: contextual observations are clearly organized by domain; no standalone qualitative theme hierarchy is claimed. |
| 32 | Clarity of minor themes: Is there a description of diverse cases or discussion of minor themes? | Not reported as a standalone qualitative analysis. | Not applicable / not reported: the qualitative strand is used for selected contextual observations rather than a full major/minor theme structure. |

**CHERRIES: the Checklist for Reporting Results of Internet E-Surveys**

Study title: AI Literacy and Training Needs Among French Midwifery Students: National Cross-Sectional Online Survey and Exploratory Qualitative Follow-Up

Eysenbach G. Improving the quality of Web surveys: the Checklist for Reporting Results of Internet E-Surveys (CHERRIES). J Med Internet Res. 2004 Sep 29;6(3):e34. doi: 10.2196/jmir.6.3.e34. Erratum in: doi:10.2196/jmir.2042. PMID: 15471760; PMCID: PMC1550605.

| **Item category** | **Checklist item** | **Explanation** |
| --- | --- | --- |
| **Design** | Describe survey design | National cross-sectional, web-based survey of midwifery students in France; voluntary convenience sample recruited through institutional and student-association channels; complemented by an exploratory qualitative follow-up strand (reported via COREQ). |
| **IRB approval & consent** | IRB approval | Approved by the Ethics Committee for Integrity and Research in Health Professions Education of the International Francophone Society for Medical Education; (SIFEM); notice n° 1210-2023, approval date 26 November 2023. |
|  | Informed consent | An electronic information sheet was presented before access to the questionnaire and electronic consent was obtained before completion. An estimated completion time was also displayed. |
|  | Data protection | No directly identifying information was collected. Survey data were stored securely, with access restricted to the research team. |
| **Development & pre-testing** | Development and testing | Items were drawn from five previously published instruments or questionnaires with heterogeneous prior psychometric evaluation; full French versions were cross-culturally translated, adapted, and linguistically validated [Kotzki 2026]. Technical/usability pre-testing of the assembled survey was not documented. |
| **Recruitment / sample access** | Open vs closed survey | Open in the technical sense (link not individualized, not password-protected), but restricted in practice to midwifery students because the link was disseminated locally to their own students. |
|  | Contact mode | Initial contact was made via the Internet, through institutional and student-association channels. |
|  | Advertising the survey | The invitation was disseminated in several waves through institutional and student-association channels, with up to two reminders. |
|  | Web/E-mail | Web-based questionnaire hosted on a secure online survey platform; responses captured automatically. |
| **Survey administration** | Context | The survey was not hosted or promoted on a thematic website or online community; it was delivered via a secure platform and disseminated through institutional and student-association channels. Selection bias relates primarily to voluntary participation among invited students rather than to website content. |
|  | Mandatory/voluntary | Voluntary and uncompensated. |
|  | Incentives | None offered. |
|  | Time/Date | Data collected between January 1^st^ and May 31^st^ 2025. |
|  | Randomization of items | Not reported. |
|  | Adaptive questioning | Not reported. |
|  | Number of items | 112 items in total: 94 Likert-type items, 13 single-choice items, and 5 open-ended items. |
|  | Number of screens (pages) | Distributed over five pages/screens (sociodemographic items first, followed by the AI-related sections). |
|  | Completeness check | No pre-submission completeness check was documented. Partial responses were automatically saved, and only fully completed questionnaires were retained for analysis. |
|  | Review step | Not reported. |
| **Response rates** | Unique site visitor | Not measurable: the survey was disseminated by email/local channels and not embedded in a single website with stable traffic denominators. |
|  | View rate | Not available. |
|  | Participation rate | Not available. |
|  | Completion rate | Completion was reported as 190 completed questionnaires among 414 initiated survey entries (45.9%). |
| **Preventing multiple entries** | Cookies used | Not used (no duplicate-entry prevention, to preserve complete anonymity). |
|  | IP check | Not used. |
|  | Log file analysis | Not performed. |
|  | Registration | Not applicable (no login/registration). |
| **Analysis** | Handling of incomplete questionnaires | Only fully completed questionnaires were analyzed; partially completed entries were excluded (complete-case analysis, n=190; no imputation). |
|  | Questionnaires with an atypical timestamp | No completion-time cutoff was applied. |
|  | Statistical correction | No weighting or propensity-score adjustment was applied; descriptive complete-case analysis only. |
